# Aβ mediates F-actin disassembly in dendritic spines leading to cognitive deficits in Alzheimer's disease

**DOI:** 10.1523/JNEUROSCI.2127-17.2017

**Published:** 2018-01-31

**Authors:** Reddy Peera Kommaddi, Debajyoti Das, Smitha Karunakaran, Siddharth Nanguneri, Deepti Bapat, Ajit Ray, Eisha Shaw, David A. Bennett, Deepak Nair, Vijayalakshmi Ravindranath

**Affiliations:** ^1^Centre for Neuroscience, Indian Institute of Science, Bangalore 560012, India,; ^2^Centre for Brain Research, Bangalore 560012, India, and; ^3^Rush Alzheimer's Disease Center, Rush University Medical Center, Chicago, Illinois 60612

**Keywords:** cognition, cytoskeleton, dementia, neurodegenerative disease

## Abstract

Dendritic spine loss is recognized as an early feature of Alzheimer's disease (AD), but the underlying mechanisms are poorly understood. Dendritic spine structure is defined by filamentous actin (F-actin) and we observed depolymerization of synaptosomal F-actin accompanied by increased globular-actin (G-actin) at as early as 1 month of age in a mouse model of AD (APPswe/PS1ΔE9, male mice). This led to recall deficit after contextual fear conditioning (cFC) at 2 months of age in APPswe/PS1ΔE9 male mice, which could be reversed by the actin-polymerizing agent jasplakinolide. Further, the F-actin-depolymerizing agent latrunculin induced recall deficit after cFC in WT mice, indicating the importance of maintaining F-/G-actin equilibrium for optimal behavioral response. Using direct stochastic optical reconstruction microscopy (dSTORM), we show that F-actin depolymerization in spines leads to a breakdown of the nano-organization of outwardly radiating F-actin rods in cortical neurons from APPswe/PS1ΔE9 mice. Our results demonstrate that synaptic dysfunction seen as F-actin disassembly occurs very early, before onset of pathological hallmarks in AD mice, and contributes to behavioral dysfunction, indicating that depolymerization of F-actin is causal and not consequent to decreased spine density. Further, we observed decreased synaptosomal F-actin levels in postmortem brain from mild cognitive impairment and AD patients compared with subjects with normal cognition. F-actin decrease correlated inversely with increasing AD pathology (Braak score, Aβ load, and tangle density) and directly with performance in episodic and working memory tasks, suggesting its role in human disease pathogenesis and progression.

**SIGNIFICANCE STATEMENT** Synaptic dysfunction underlies cognitive deficits in Alzheimer's disease (AD). The cytoskeletal protein actin plays a critical role in maintaining structure and function of synapses. Using cultured neurons and an AD mouse model, we show for the first time that filamentous actin (F-actin) is lost selectively from synapses early in the disease process, long before the onset of classical AD pathology. We also demonstrate that loss of synaptic F-actin contributes directly to memory deficits. Loss of synaptosomal F-actin in human postmortem tissue correlates directly with decreased performance in memory test and inversely with AD pathology. Our data highlight that synaptic cytoarchitectural changes occur early in AD and they may be targeted for the development of therapeutics.

## Introduction

Alzheimer's disease (AD) is a progressive neurodegenerative disorder that is characterized by impairment of cognitive functions, including memory. Longitudinal studies in humans have revealed that accumulation of β-amyloid, thinning of the cortex, and decreased hippocampal volume precedes cognitive dysfunction. At the cellular level, the biological signatures of AD are synaptic dysfunction including synapse loss, β-amyloid plaques, hyperphosphorylated tau, and extensive neurodegeneration. Synaptic dysfunction seen as loss of dendritic spines in mouse models of AD (Wu et al., 2010; Herms and Dorostkar, 2016) and that observed as decreased glucose utilization (FDG-PET imaging) in human subjects precedes the overt appearance of behavioral/cognitive dysfunction (Jack and Holtzman, 2013; Jack et al., 2013). Loss of dendritic spines is seen early in disease pathogenesis in mouse models of AD (Spires-Jones et al., 2007) and in postmortem brains from AD patients (Tsai et al., 2004; Spires et al., 2005; Spires-Jones et al., 2007). However, the mechanisms underlying synaptic dysfunction, including loss of spines, is not well understood.

Dendritic spines along neurites are the primary sites for receiving information and cellular substrates for synaptic plasticity. Loss of spines often results in defective synaptic transmission (Cummings et al., 2015). Dendritic spines undergo synaptic-activity-dependent modifications such as enlargement and shrinkage/elimination during LTP (Bosch et al., 2014) or LTD, respectively, and correlate with memory deficits in animal models (Holcomb et al., 1998; Lesné et al., 2006; Shankar et al., 2008). Filamentous actin (F-actin) is the major cytoskeletal protein in spines and spine structure is regulated by remodeling of actin cytoskeleton, including those that occur during stabilization of memories after learning (Dillon and Goda, 2005; Frost et al., 2010; Hotulainen and Hoogenraad, 2010; Chazeau et al., 2014; Efimova et al., 2017).

Actin exists in two states: as monomeric globular-actin (G-actin), which polymerizes to form asymmetric two-stranded helical F-actin. Polymerization and depolymerization of actin (actin treadmilling; F-actin/G-actin ratio) regulates various features of dendritic spine morphology (Okamoto et al., 2007; Hotulainen et al., 2009). F-actin is highly enriched in dendritic spines and dynamic modulation of this protein is critical for controlling, not only spine formation and elimination, but also synaptic-activity-dependent structural changes in dendritic spines (Star et al., 2002; Okamoto et al., 2007; Korobova and Svitkina, 2010). Therefore, maintaining the ratio of F-actin and G-actin within the dendritic spines is essential for optimal synaptic function.

Change in the ratio of F- and G-actin is regulated by different actin-interacting molecules, such as ADF/cofilin (Bamburg et al., 2010; Bernstein and Bamburg, 2010; Gu et al., 2010; Bamburg and Bernstein, 2016), Cdc42 (Heredia et al., 2006; Mendoza-Naranjo et al., 2012), mTORC2 (Huang et al., 2013), LIMK1 (Heredia et al., 2006), and Mical (Hung et al., 2013), as well as through posttranslational modifications such as acetylation, phosphorylation, methylation, and redox modifications of cysteine thiols (Terman and Kashina, 2013).

We investigated whether shifting of the F-/G-actin equilibrium toward G-actin could underlie the loss of spines seen in AD, rather than that occurring as a consequence of spine loss. We used three model systems, APPswe/PS1ΔE9 mice (APP/PS1; both presymptomatic young mice and middle-aged animals), primary neurons derived from WT and APP/PS1 mice, and primary neurons derived from C57BL/6 mice and exposed to low concentrations of Aβ_42_ peptide. We assayed total actin and F-/G-actin levels in synaptosomes prepared from cortex of adolescent (ADL) AD mice (1 month old) because synaptic dysfunction is considered to occur before the onset of cognitive dysfunction and we used primary neurons from WT and APP/PS1 mice to examine the nanoscale organization of F-actin in spines. To determine whether our observations can be extrapolated to the human disease, we also performed experiments using human postmortem tissue from subjects with no cognition impairment (NCI), mild cognitive impairment (MCI), and AD.

## Materials and Methods

### 

#### 

##### Experimental design.

We hypothesized that Aβ-induced F-actin depolymerization may lead to spine loss in AD and that this occurs before the overt onset of the disease. We assayed the synaptosomal levels of F-actin, G-actin, and total actin in postmortem frontal cortex from NCI, MCI, and AD subjects. We used frontal cortex samples from 12 subjects per group considering inherent variability between individual samples in terms of genetic and nongenetic differences and disease pathology, which is further compounded by differences in postmortem interval. All *n*'s were used for regression and general linear model (GLM) analysis.

We also performed experiments (both behavioral and biochemical) *in vivo* using APP/PS1 (male) and WT mice (male) and mixed-sex primary cortical neurons to study structural deficits including F-actin nanoarchitecture in spines. For confocal microscopy, sample size included primary neurons from three independent litters and 22–30 neurons from each group. For experiments using direct stochastic optical reconstruction microscopy (dSTORM), sample size was 30–35 dendritic spines from three independent litters for each group. It is known that observed power of analysis is inversely related to observed *p*-value. Transgenic expression of APP/PS1 is variable across animals, which results in variability in the measured biochemical parameters. To account for this effect and to exclude the possibility of litter-specific effects, we chose 6–10 animals per group in each biochemical experiment from different litters and processed them individually. For the behavioral studies, the number of animals to be used was based on behavioral experiments performed in several laboratories across the world and a sample size of 9–11 mice per genotype or treatment was used.

##### Data inclusion and exclusion.

No samples were excluded from any of the experiments or analyses described herein.

##### Randomization and blinding.

All of the animal experiments were designed and followed in compliance with the Animal Research: Reporting of In Vivo Experiments (ARRIVE) guidelines, including control groups for all experiments and applying double-blinded analysis when possible. In experiments involving WT and APP/PS1 mice, the animals were assigned randomly to the respective groups based on the genotype. In experiments using jasplakinolide or latrunculin, mice of particular genotypes were assigned to groups using randomization.

##### Reagents.

The following chemicals and reagents were used: G-Actin/F-actin *In Vivo* Assay Biochem Kit (catalog #BK037; Cytoskeleton), Actin Polymerization Biochem Kit (catalog #BK003; Cytoskeleton), and NeuroTrace DiI Tissue-Labeling Paste (catalog #N22880), Jasplakinolide (catalog #J7473), latrunculin A (catalog #L12370) were all from Thermo Fisher Scientific. DNase I (catalog #D-4513) and papain (catalog #P-4762) were from Sigma-Aldrich. All other chemicals and reagents were of analytical grade and were from Sigma-Aldrich.

##### Antibodies.

Antibody against β-actin was from MP Biomedicals (catalog #0869100); Acti-Stain 488 (catalog #PHDG1) was from Cytoskeleton; antibody to Drebrin (catalog #ab60933; RRID:AB_10675963) was from Abcam; PSD95 (catalog #ab18258; RRID:AB_444362) was from Abcam; Homer1 (catalog #160 003, RRID:AB_887730) was from Synaptic Systems; cofilin (catalog #5175; RRID:AB_10622000), phospho-cofilin (Ser3) (catalog #3313; RRID:AB_2080597), and Arp2 (catalog #3128, RRID:AB_2181763) were from Cell Signaling Technology); Arp3 (catalog #4738, RRID:AB_2221973) was from Cell Signaling Technology; GluA1 (catalog #13185) was from Cell Signaling Technology; and anti-β-amyloid, 1–42 antibody (catalog #805503; RRID:AB_2564682) was from BioLegend. Horseradish peroxidase-conjugated secondary antibodies were from Vector Laboratories. Alexa Fluor 647 Phalloidin (catalog #A22287; RRID:AB_2620155) was from Thermo Fisher Scientific.

##### Postmortem brain tissues.

Frontal neocortical tissue from participants in the Religious Orders Study conducted by the Rush Alzheimer's Disease Center (Chicago, IL) was obtained. All participants signed an informed consent and an Anatomical Gift Act for brain donation. The study was approved by the Institutional Review Board of Rush University Medical Center and the Indian Institute of Science. All experiments involving human postmortem tissues were performed in accordance with institutional guidelines and after approval from the ethics committee. Details of the study and the diagnostic approach and neuropathological assessments have been described previously (Bennett et al., 2002; Wilson et al., 2002; Bennett et al., 2004, 2005, 2006, 2012). A total of 36 brains were examined: 12 NCI cases, 12 MCI cases, and 12 AD cases for comparison. Frontal cortical tissue from each human brain was thawed on ice and used for the preparation of postnuclear supernatant (PNS) and synaptosomes as described below. All fractions were aliquoted and stored at −86°C.

##### Experimental animals.

Transgenic mice B6C3-Tg (APP_Swe_/PS1ΔE9)85Dbo/J (https://www.jax.org/strain/005864) were from The Jackson Laboratory. WT and APPswe/PS1ΔE9 (APP/PS1) mice were bred at the Institutional Central Animal Facility and 1 month-old (30–35 d; ADL), 2-month-old (60–70 d), 4-month-old (120–130 d), or 9-month-old (270–300 d; middle-aged; MA) male mice were used for the experiments. Animals were housed under pathogen-free conditions in a temperature-controlled room on 12 h light/12 h dark cycle and had *ad libitum* access to food and water. All animal experiments were performed in accordance with institutional guidelines for the care and use of laboratory animals under approval of the Institutional Animal Ethics Committee and effort was made to reduce suffering of animals and the numbers used.

##### Contextual fear conditioning (cFC).

All experiments were performed with 2-month-old male mice. The cFC training context was rectangular in shape. Identity of the context was maintained with the presence of distinct odor (2% acetic acid, v/v). The conditioning chamber was cleaned with 70% ethanol before and after each session. Mice were single housed and were first handled for 5 min for 3 d. On training day, mice were allowed to explore the training context for 1 min and then received 3 foot shocks (2 s and 0.6 mA each, intertrial interval 30 s). Contextual fear memory was assessed by returning mice to the training context 24 h after fear conditioning and analyzing freezing during a test period of 2 min. Freezing was defined as complete absence of somatic mobility other than respiratory movements. No animals were excluded from the analysis. In some experiments, jasplakinolide or latrunculin A (Invitrogen) was freshly dissolved in DMSO (3% in normal saline; 100 μl) and then injected intrathecally at a dose of 0.5 μg/mice immediately after training.

##### Assay of G-actin and F-actin.

Synaptosomes were prepared as described previously (Ahmad et al., 2017) and resuspended in lysis buffer supplemented with 1 mm ATP and protease inhibitor mixture for F-actin stabilization (Cytoskeleton). For preparation of G-actin and F-actin fractions from PNS, cortical tissue was homogenized directly in the above buffer and centrifuged at 1500 × *g* for 10 min. Further, G-actin and F-actin fractions were separated using the G-Actin/F-Actin *In Vivo* Assay Kit according to the manufacturer's instructions (Cytoskeleton, catalog #BK037). Protein concentrations were determined using the Pierce BCA protein assay kit before immunoblotting. All samples were resolved using TGX Stain Free Fast Cast Acrylamide kit (12%; Bio-Rad) and transferred onto a PVDF membrane for immunoblotting. Stain-free blots were imaged before antibody incubation using Bio-Rad Chemidoc-XRS and analyzed with Image lab software (Bio-Rad). Stain-free gels/blots showed better staining, so the stain-free detection method was used as a loading control instead of housekeeping proteins for unbiased normalization (Colella et al., 2012; Gilda and Gomes, 2013; Gürtler et al., 2013; Rivero-Gutiérrez et al., 2014; Gilda and Gomes, 2015). Immunoreactive bands were detected using enhanced chemiluminescence (Clarity Western ECL blotting substrate; Bio-Rad). Signals were detected (Bio-Rad Chemidoc-XRS) and analyzed with Imagelab software (Bio-Rad).

##### Actin polymerization assay.

**Figure 1.**
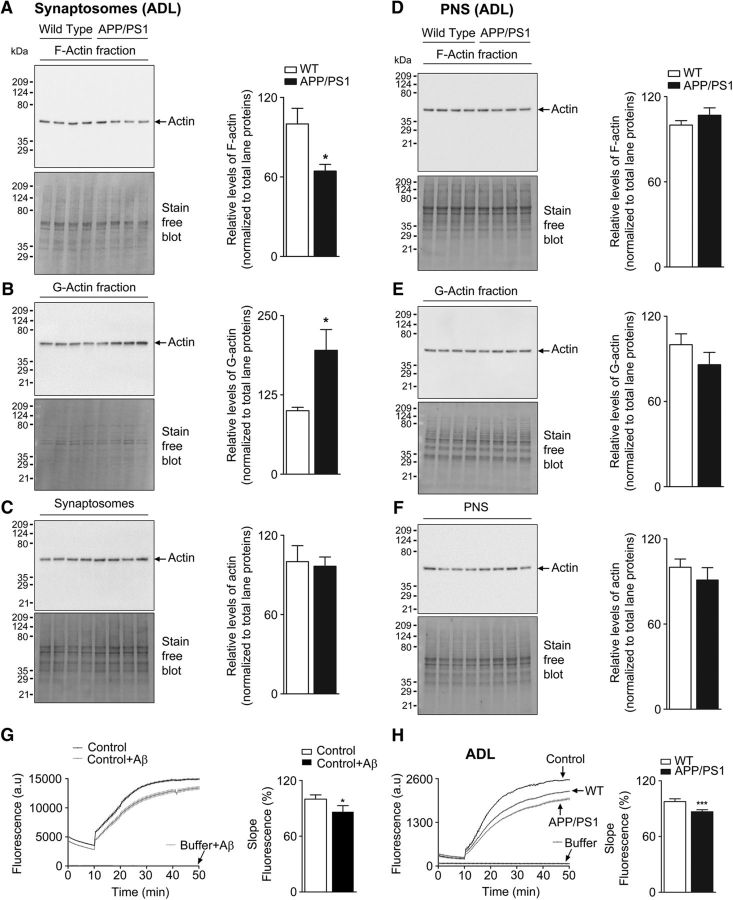
Decreased synaptosomal F-actin levels in cerebral cortex of APP/PS1 mice (see Fig. 1-1, Fig. 1-2, Fig. 1-3). Enriched G-actin and F-actin fractions were isolated from synaptosomes and PNS of ADL (1-month-old) WT and APP/PS1 mouse brain cortex samples. These fractions were resolved on TGX-stain-free gels and levels of F-actin (***A***, ***D***), G-actin (***B***, ***E***), and total actin (***C***, ***F***) were analyzed by immunoblotting with anti-actin antibody. Densitometric analyses for actin levels were normalized to total lane proteins. Statistical analysis: ***A***, ADL mice quantification of F-actin (*p* = 0.0144, *t* = 2.792, df = 14); ***B***, G-actin (*p* = 0.0125, *t* = 2.865, df = 14); ***C***, total actin (*p* = 0.8089, *t* = 0.2465, df = 14); ***D***, ADL mice quantification of F-actin (*p* = 0.2710, *t* = 1.146, df = 14); ***E***, G-actin (*p* = 0.2438, *t* = 1.217, df = 14); ***F***, total actin (*p* = 0.4030, *t* = 0.8624, df = 14). Unpaired two-tailed *t* test was used. Results are shown as the mean ± SEM (*n* = 7–8 per group). ***G***, *In vitro* actin polymerization assay was performed using control actin only and control actin reaction supplemented with 62.5 nm Aβ_1-42_ peptide (*p* = 0.0401, *t* = 2.996, df = 4). Results are shown from three experiments. Unpaired two-tailed *t* test was used. Asterisks indicates statistical significance. ***H***, Rate of actin polymerization was measured from synaptosomes of ADL mice. For actin polymerization, results are shown from ADL (*n* = 8) mice per group. Statistical significance: *p* < 0.0001, *t* = 8.379, df = 14. *p < 0.05, ***p < 0.001 for WT versus APP/PS1 (unpaired Student's two-tailed *t* test).

**Figure 2.**
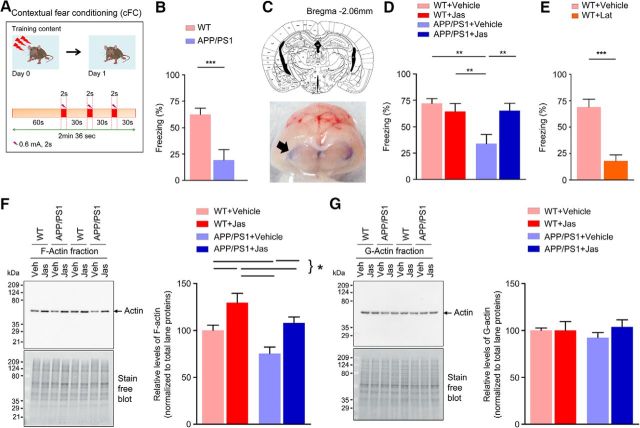
Long-term fear memory is impaired in 2-month- old APP/PS1 mice (see Fig. 2-1). ***A***, Schematic of the experimental design. ***B***, APP/PS1 mice exhibit significantly reduced freezing response than the WT control (*p* = 0.0021, *t* = 3.633, df = 17; *n* = 9–10 per group). ***C***, Analysis of dye spread. Top, Schematic drawing based on the atlas of Franklin and Paxinos (1997) of coronal section of mouse brain depicting the location of cerebral ventricles (3V, D3V, LV), along with anteroposterior coordinates caudal to bregma. Bottom, Coronal cross-section of the brain harvested 4 h after intrathecal dye injection (100 μl of 1% methylene blue solution) to illustrate localization of dye spread in the cerebral ventricles (arrow). ***D***, Intrathecal delivery of jasplakinolide (Jas) or vehicle after cFC rescued fear memory in APP/PS1 mice (interaction: *F* = 7.34482, *p* = 0.0101, df = 37; Jas: *F* = 2.77348, *p* = 0.1043, df = 37; genotype: *F* = 6.76938, *p* = 0.0133, df = 37; *n* = 9–11 per group). ***E***, Reduced conditioned freezing levels were observed in WT control mice after intrathecal delivery of vehicle or latrunculin A (Lat) after training (*p* < 0.0001, *t* = 5.411, df = 16).(*n* = 9 per group). ***F***, ***G***, Representative immunoblot analysis for the synaptosomal F-actin and G-actin fractions in samples collected 24 h after injection of vehicle or Jas. Quantification of F-actin (interaction: *F* = 0.0443737, *p* = 0.8347, df = 28; Jas: *F* = 17.7623, *p* = 0.0002, df = 28; Genotype: *F* = 9.80714, *p* = 0.0040, df = 28; *n* = 7–9 per group). Quantification of G-actin (interaction: *F* = 0.686628, *p* = 0.4143, df = 28; Jas: *F* = 0.676441, *p* = 0.4178, df = 28; genotype: *F* = 0.0813757, *p* = 0.7775, df = 28; *n* = 7–9 per group). Statistical comparison was performed using unpaired two-tailed *t* test (***B***, ***E***) or two-way ANOVA followed by Newman–Keuls post test (***D***, ***F***, ***G***). Data are presented as mean ± SEM. Statistical significance: *p < 0.05,**p < 0.01,***p < 0.001.

The actin polymerization assay was performed with or without the presence of Aβ_1-42_ peptide (62.5 nm). The assay was also performed in the presence of synaptosomes isolated from WT and APP/PS1 mice (see Fig. 1*G*,*H*) according to the described protocol in the actin polymerization biochem kit (catalog #BK003; Cytoskeleton). The actin polymerization assay was also performed in the presence of increasing concentrations of G-actin protein (catalog #AKL99, Cytoskeleton; Fig. 1-2*A*). Furthermore, WT synaptosomes were incubated with or without Aβ_1-42_ (62.5 nm) for 1 h at 37°C. After incubation, synaptosomes were used for the actin polymerization assay, which was performed according to the manufacturer's instructions (Fig. 1-1*B*,*C*).

##### Immunolabeling of Aβ_42_ aggregates in APP/PS1 mice.

Paraffin-embedded sections of WT and APP/PS1 mouse brain were prepared. Sections were dewaxed and transferred to PBS containing hydrogen peroxide (3% v/v) to block the endogenous peroxidase reaction. The sections were cooked in a pressure cooker using sodium citrate buffer (0.01 m, pH 6) for antigen retrieval, blocked with normal goat serum, and incubated with anti-β-amyloid, 1–42 antibody. The sections were washed, treated with biotinylated anti-mouse-IgG, and incubated with VECTASTAIN-Elite ABC reagent (Vector Laboratories). Color was developed using Novared (ImmPact NovaRED; catalog #SK-4805; Vector Laboratories) and hydrogen peroxide. The sections were washed in water, dehydrated in graded ethanol, cleared with xylene, dried, and mounted in DPX. Images were captured using an Olympus BX83 microscope under a 10× objective.

##### Primary cortical neuronal culture.

Mixed sex primary cortical neurons were prepared from postnatal day 0 (P0) or P1 pups from both WT and APP/PS1 mice and from C57BL/6 mice. Cultures were established and maintained according to a previously published protocol (Beaudoin et al., 2012). Cortical neurons were seeded on coverslips precoated with poly-d-lysine (0.1 mg/ml). Neurobasal A medium supplemented with B27, 2 mm l-GlutaMAX, and 100 μg/ml penicillin/streptomycin was used to grow the cells in serum-free conditions and maintained at 37°C in 5% CO_2_ for 2–3 weeks.

Primary cortical neurons were fixed with 2% paraformaldehyde (w/v) and labeled with DiI or Actin-Stain 488 Phalloidin (Cytoskeleton) according to the manufacturer's instructions. Neurons were also stained with antibody against actin or cofilin, followed by secondary antibody.

For dSTORM imaging, samples were fixed with 0.3% glutaraldehyde (v/v) and blocked with 3% BSA (w/v) containing 0.2% Triton X-100 (v/v) for 30 min. Homer1 antibody was used for immunostaining, followed by Alexa Fluor 532 secondary antibody. F-actin labeling was then performed with Alexa Fluor 647–phalloidin (Xu et al., 2013).

##### Treatment of cells with rhodamine-tagged Aβ_42_.

Rhodamine labeled Aβ_42_ was synthesized by coupling 5-(6) tetramethyl rhodamine carboxylic acid with the N terminus (Asp1) of Aβ_42_ and was obtained as a gift from Prof. Sudipta Maiti (Tata Insititute of Fundamental Research, Mumbai, India). The lyophilized peptide was resuspended in NaOH solution, pH 11, to a final concentration of 1 mm. The required concentration of rhodamine-tagged Aβ_42_ used in the experiments was prepared by serially diluting the peptide using culture medium. After 24 h of treatment, the medium was removed and cells were washed twice with warm PBS before fixation.

##### Image acquisition and analysis.

Confocal images were acquired using a Carl Zeiss LSM780 laser scanning system with an argon 488 laser for DiI-514 and Acti-Stain 488 Phalloidin. A helium–neon 594 laser was used to visualize rhodamine-Aβ_1-42_. An oil-immersion objective 63×/1.40 numerical aperture (NA) was used and *z*-stack images were captured using the following parameters: 512 × 512 resolution and 12 bit depth, zoom factor of 3 (except for rhodamine-Aβ_1-42_, for which the zoom factor was 1), pinhole 1 airy unit, and step size interval of 0.4 μm. All confocal images for DiI- and phalloidin-labeled neurites were acquired under identical conditions as described above and analyzed after blinding.

**Figure 3.**
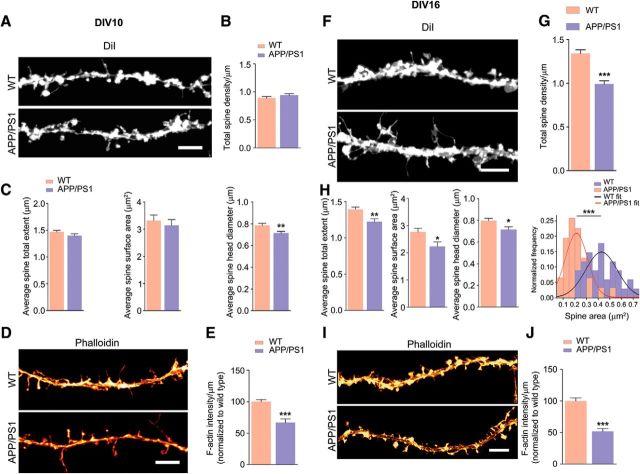
Loss of total dendritic spines, spine total extent, spine surface area, spine head diameter, and F-actin in APP/PS1 primary cortical neurons. ***A***, ***F***, Primary cortical neurons were fixed at DIV 10 and DIV 16 and analyzed. Representative confocal images of dendritic spines were visualized by DiI labeling. ***B, C***, Quantitative dendritic spine analysis: ***B***, DIV 10; total spine density, *p* = 0.2056, *t* = 1.283, df = 48; ***C***, Spine total extent, *p* = 0.1654, *t* = 1.409, df = 47; spine surface area, *p* = 0.5573, *t* = 0.5911, df = 47; spine head diameter, *p* = 0.0063, *t* = 2.860, df = 47. ***D***, Confocal images showing F-actin staining of tertiary dendrite using phalloidin labeling. Scale bar, 5 μm. ***E***, F-actin quantification (DIV 10; *p* < 0.0001, *t* = 5.162, df = 62). ***G***, DIV 16 total spine density (*p* < 0.0001, *t* = 6.244, df = 58; ***H***, spine total extent (*p* = 0.0017, *t* = 3.283, df = 58; spine surface area, *p* = 0.0166, *t* = 2.468, df = 58; spine head diameter, *p* = 0.0162, *t* = 2.477, df = 58). Cross-sectional area of 2D maximum intensity projections of spines (expressed as square micrometers) were plotted and fitted by normal distribution curve; the fit generated WT mean = 0.427, variance = 0.018; APP/PS1 mean = 0.213, variance = 0.009. Using unpaired two-tailed *t* test, the statistical significance is as follows: *p* < 0.0001, *t* = 7.293, df = 63. ***I***, Confocal images showing F-actin staining of tertiary dendrite using phalloidin. Scale bar, 5 μm. ***J***, F-actin quantification (DIV 16; *p* < 0.0001, *t* = 7.025, df = 62). ***E***, ***J***, Phalloidin (F-actin) quantification was performed from two tertiary neurites (similar lengths of dendrites including spines) from single neuron. Data are represented as mean ± SEM from three independent experiments. *n* = 30–34 neurites (5–6 neurons and 10–12 tertiary neurites from each independent experiment). Statistical significance: *p < 0.05,**p < 0.01,***p < 0.001. Statistical comparisons were performed using unpaired two-tailed *t* test.

DiI-labeled neurons were used for spine analysis and analysis was performed using Neurolucida 360 as described previously (Dickstein et al., 2016). *z*-stack images captured using a confocal microscope were loaded into Neurolucida 360 and maximum intensity projections (MIPs) were generated for each bit of dendrite. The backbone of the dendritic branch was traced and the dendrite was modeled accurately in all three dimensions. Spine-related parameters on each dendritic branch were automatically quantitated by the software using spine detection mode. The reports include total number and spine density per micrometer and the other details such as spine total extent, spine surface area, and spine head diameter. In addition, the cross-sectional area of 2D MIPs was measured for assessing the spine area (see Fig. 3*H*) and its histogram was plotted and fitted with normal distribution using the distribution fitting function from Statistics tool box version 10 of MATLAB R2015a (The MathWorks).

Quantification of F-actin levels measured as phalloidin intensity was performed using MetaMorph software (version 7.8.0.0, 2013; Molecular Devices). Confocal *z*-stack images were loaded onto MetaMorph software and MIPs were generated. After background subtraction and thresholding, a mask was generated around the dendrite of interest (including the spines) and phalloidin intensity was measured.

##### dSTORM imaging and analysis.

Samples were imaged at 37°C (OKO lab, Italy) in a closed chamber used for loading 18 mm round coverslips (Ludin Chamber; Life Imaging Services) and mounted on an inverted motorized microscope (Olympus IX83) equipped with a 100×, 1.49 NA PL-APO objective and an azimuthal drift control device, allowing long acquisition in oblique illumination mode using multilaser launch (Roper). The images were acquired at the center quadrant (256 × 256 pixel^2^) of an EMCCD camera (Evolve; Photometrics). The illumination and acquisition was controlled by MetaMorph. Before imaging, postfixation was performed with 4% paraformaldehyde (w/v). Beads of 100 nm diameter (Tetraspeck; Thermo Fisher Scientific) were used as fiduciary markers for lateral drift correction. Immunolabeled cells were imaged in a dSTORM buffer with a mixture of chemicals to induce stochastic activation of sparse subsets of molecules (catalase, TCEP, glycerine, glucose and glucose oxidase dissolved in Tris-HCl buffer). dSTORM buffer was added before imaging (Nair et al., 2013; Chazeau et al., 2014). Photoconversion of carbocyanine dyes from ensemble to single-molecule density was achieved by illuminating the sample with a 300 mW excitation laser to convert fluorescent molecules into the metastable dark state. After achieving an optimal density of 0.01–0.04 molecules/μm^2^, the illumination laser power was optimized to 150 mW and 5 stacks of 4000 frames each were obtained each with an exposure time of 20 ms, acquiring a total of 20,000 images. The instantaneous densities of single molecules were controlled by modulating the power of 405 nm laser.

Image acquisition, processing, and subsequent analysis was performed using a custom based module optimized within MetaMorph (Izeddin et al., 2012; Nair et al., 2013; Venkataramani et al., 2016). The localization accuracy of the optical system was determined from the localization of centroids of point spread functions generated by 100 nm fluorescent beads. The fluorescent beads were imaged for 4000 frames at similar laser intensities recorded for the dSTORM measurements on immunolabeled samples (Fig. 5-1*A*,*B*). The centroid of the lateral spread of the fluorescence intensity was calculated for every frame and accuracy of detection histogram was obtained for all the localization in time (Fig. 5-1*A*). The resulting spread was modeled using a bidimensional Gaussian function providing a localization precision (σ) of ∼19.4 nm (Fig. 5-1*C*). The full width at half maximum was calculated to be ∼44.6 nm (Fig. 5-1*D*) (Nanguneri et al., 2012).

After dSTORM reconstruction of raw images, random mushroom spines were selected for automated analysis of radiating actin structures within the spine head using an ImageJ plugin called Ridge Detection (Steger, 1998). In addition to the detection of these rods, we were able to automatically quantify the orientation of the radiating structures using another ImageJ plugin called FibrilTool (Boudaoud et al., 2014). FibrilTool calculates a value referred to as “anisotropy,” which is a measure of how parallel the fibers are with respect to each other. Mean anisotropy values were compared between spines from WT and APP/PS1 mice to quantify changes in F-actin organization within spine head.

##### GLM analyses.

Ordinary least-squares regression-based GLMs were analyzed to understand the impact of clinical histopathological scores (Braak staging, β-amyloid load, and tangle density) on F-actin or G-actin expression. To control for skewness in β-amyloid load and tangle density distribution, GLM was performed with the square root values of the respective distributions. In these GLMs, the predictor variables included different histopathological scores, along with age at death and postmortem interval as confounding variables, whereas the dependent variable was either F-actin or G-actin expression. GLMs were also performed for assessing whether F-actin or G-actin expression predicted the subject's last measured cognitive performance when controlled for confounding variable of educational level and age at death. Cognitive performance was measured as global cognition (Cogn_Glob), episodic memory (Cogn_Epi), working memory (Cogn_WM), semantic memory (Cogn_Sem), perceptual speed (Cogn_PS), or perceptual orientation (Cogn_PO). Cogn_Glob is a composite score derived by pooling all of the scores from 19 tests that are used to generate the individual cognitive domains. All cognitive scores were converted into *z*-scores for analyses based on mean and SDs of the entire cohort being studied. Because several of these parameters are correlated with each other, we did not consider variable interaction terms in our GLM analyses. A significant *p*-value in these analyses indicates relative predictive strength of individual predictor variables upon the dependent variable after controlling for confounding variables.

##### Statistical analyses.

Statistical differences between two groups were performed with two-tailed unpaired Student's *t* test and those between more than two groups were performed with one-way ANOVA followed by Newman–Keuls *post hoc* test of multiple comparisons. Two-way ANOVA with Newman–Keuls *post hoc* test was used for experiments with four groups. Results are represented as mean ± SEM. *p*-values <0.05 were regarded as significant. Graphs and statistics were performed using GraphPad Prism software (Version 5 or 6).

## Results

### Depolymerization of synaptosomal F-actin in cerebral cortex of 1-month-old AD mice

We used mouse model of AD (APPswe/PS1ΔE9) to examine potential disassembly of F-actin in dendritic spines. To address this, we assessed F-actin, G-actin, and total actin levels in synaptosomes and PNS from the cortex of 1-month-old (ADL) male AD mice; at this age, the mice do not display pathological hallmarks of the disease. Remarkably, ADL APP/PS1 mice showed a significant decrease in synaptosomal F-actin levels compared with WT littermate controls (Fig. 1*A*). This was accompanied by corresponding increase in G-actin levels (Fig. 1*B*), indicating that F-actin was depolymerizing to G-actin without any change in total actin concentration. The loss of F-actin was seen selectively in synaptosomes but not in PNS (Fig. 1*D*).

10.1523/JNEUROSCI.2127-17.2017.f1-1 Figure 1-1 Effect of Aβ_1-42_ on the assembly of linear actin filaments in vitro (Refer to Figure 1 in the main article). (A) Rate of actin polymerization was measured in the absence or presence of increasing concentrations of G-actin standard protein. (B-C) Wild type synaptosomes were pretreated with or without Aβ_1-42_ to give final concentration of 62.5 nM and the samples were incubated at 37°C for 1 hour. After incubation, rate of actin polymerization was measured (p=0.0130, t=4.263, *df*=4). Unpaired two-tailed t-test. * indicates statistical significance. Download Figure 1-1, TIF file

10.1523/JNEUROSCI.2127-17.2017.f1-2 Figure 1-2 Biochemical analyses of actin expression in synaptosomes, PNS, F-actin and G-actin fractions (Refer to Figure 1 in the main article). Isolated F-actin and G-actin fractions from synaptosomes (MA; A-C) and post nuclear supernatant (MA; D-F) of middle aged wild type and APP/PS1 mouse brain cortex samples. These fractions were resolved on stain free gels and immunoblotted against actin antibody. Densitometric analysis for actin levels from F-actin, G-actin, synaptosomes and PNS fractions were normalized to total lane proteins. Statistical analysis: (A) MA-mice-quantification of F-actin (p=0.0003, t=4.692, *df*=14); (B) G-actin (p=0.7104, t=0.3790, *df*=14); (C) actin (p=0.7287, t=0.3551, *df*=12). Results were represented as the mean ± s.e.m. (n=7-8 per group). (D) MA mice-quantification of F-actin (p=0.9398, t=0.07692, *df*=14); (E) G-actin (p=0.2768, t=1.132, *df*=14); (F) actin (p=0.0598, t=2.061, *df*=13). Results were represented as the mean ± s.e.m. (n=7-8 per group). Statistical significance: *p< 0.05, ***p< 0.001 for wild type versus APP/PS1 (Unpaired Student's two-tailed t-test). (G) Rate of actin polymerization was measured from synaptosomes of MA mice (p<0.0026, t=4.939, *df*=6). For actin polymerization, results were represented from MA (n=4) mice per group. Unpaired two-tailed t-test. * indicates statistical significance. Download Figure 1-2, TIF file

10.1523/JNEUROSCI.2127-17.2017.f1-3 Figure 1-3 Abundance of synaptic scaffold proteins (PSD95, homer1) and glutamate receptor subunits (GluA1) are not altered in synaptosomes isolated from ADL and 2M APP/PS1 mice (Refer to Figure 1 in the main article). PSD95 (A, D), Homer1 (B, E) and GluA1 (C, F) levels are not altered in synaptosomes isolated from ADL and 2M APP/PS1 mice compared to age-matched WT mice. Statistical analysis: (A) PSD95/tubulin (p=0.4786, t=0.7554, *df*=6). (B) Homer1/tubulin (p=0.1066, t=1.897, *df*=6). (C) GluA1/tubulin ((p=0.3609, t=0.9890, *df*=6). (n=4 per group). (D) PSD95/tubulin (p=0.8351, t=0.2174, *df*=6). (E) Homer1/tubulin (p=0.3499, t=1.014, *df*=6). (F) GluA1/tubulin ((p=0.2363, t=1.316, *df*=6). (n=4 per group). (n=4 per group). Unpaired two-tailed t-test. * indicates statistical significance for wild type versus APP/PS1. (G) Coronal brain sections from APP/PS1 (ADL, 2M and MA) mice were immunostained with monoclonal anti-β-amyloid, 1-42 antibody. Extracellular deposits of β-amyloid peptide seen as positively stained plaques were detected only in middle aged (MA) APP/PS1 mice. Scale bar = 100 μm. Download Figure 1-3, TIF file

We next investigated whether Aβ_1-42_ affected the kinetics of actin polymerization *in vitro*. The kinetics of actin assembly in the presence or absence of Aβ_1-42_ was performed in an assay containing pyrene–actin. Our results indicate that Aβ_1-42_ alone decreased actin dynamics (Fig. 1*G*) compared with the corresponding vehicle control (Fig. 1-1*B*,*C*). Further, the addition of WT synaptosomes alone to the pyrene–actin polymerization reaction mixture resulted in lowering of fluorescence intensity due to competition from unlabeled G-actin in the synaptosomes. This response was further amplified upon addition of synaptosomes from APP/PS1 mice (Fig. 1*H*). Together, our data indicate that selective perturbation of synaptosomal F-actin equilibrium occurs early in life, which could have profound effects on synapse function by affecting cytoskeletal architecture at the postsynaptic density during the initial stages of AD pathogenesis. The loss of synaptosomal F-actin was also seen in 9-month-old (MA) AD mice (Fig. 1-2*A*), indicating the persistent disequilibrium in F-/G-actin homeostasis.

The actin cytoskeleton is highly enriched at the postsynapse and supports scaffolding of postsynaptic specialized proteins such as PSD95 and Homer1 and clustering of glutamate receptors for efficient synaptic transmission. In view of the fact that actin regulates other synaptic proteins, such as PSD95, Homer and GluA1 receptor, we investigated whether these proteins were deregulated in APP/PS1 synaptosomes. We isolated synaptosomes from ADL and 2-month-old WT and APP/PS1 mice and performed immunoblotting. Our immunoblot analysis revealed that PSD95, homer1,and GluA1 levels were not altered at early ages (ADL and 2-month-old; Fig. 1-3*A–F*). Immunostaining of brain sections from 1-month-old (ADL), 2-month-old and 9-month-old (MA) APP/PS1 mice for Aβ42 showed substantial plaques in the cortex of 9-month-old mice, but not at 1 or 2 months of age, indicating that the pathology was absent when substantial synaptic F-actin loss is seen (Fig. 1-3*G*).

### Actin-polymerizing agent rescues impaired cFC in APP/PS1 mice

Earlier studies have demonstrated that the intact cytoskeleton provided by F-actin nanoarchitecture is required for both cued and cFC memory formation (Huang et al., 2013). We performed cFC in 2-month-old APP/PS1 mice to determine whether deficient F-actin dynamics might influence behavior. Mice were trained in cFC and tested 24 h later in the same setting. APP/PS1 mice had a significantly reduced freezing response compared with WT controls (Fig. 2*B*). To determine whether deficient actin dynamics underlies this impairment, we infused jasplakinolide, a molecule that stabilizes actin filaments, intrathecally (0.5 μg/mice) immediately after training. Jasplakinolide increased freezing response to the context in APP/PS1 mice at +24 h (Fig. 2*D*), but had no comparable effect in WT mice (Fig. 2*D*). Remarkably, 24 h after jasplakinolide administration, synaptosomal F-actin expression was restored in 2-month-old APP/PS1 mice, as seen by immunoblotting (Fig. 2*F*). The increase in F-actin levels was also seen in WT mice after jasplakinolide administration compared with vehicle controls, although no change was observed in cFC behavior (Fig. 2*F*). Further, latrunculin A, an inhibitor of actin polymerization, induced a significant decrease in freezing when infused intrathecally (0.5 μg/mice) immediately after training in 4-month-old WT mice (Fig. 2*E*). This was accompanied by decreased synaptosomal F-actin levels (Fig. 2-1*A*) compared with the vehicle controls. Together, these results provide strong evidence that perturbation of actin dynamics is involved in defective memory consolidation of context-dependent conditioned freezing in APP/PS1 mice.

10.1523/JNEUROSCI.2127-17.2017.f2-1 Figure 2-1 Long term fear memory is impaired in 2M old APP/PS1 mice (Refer to Figure 2 in the main article). (A-B) Immunoblot analysis of the synaptosomal F-actin and G-actin fractions in samples collected 24 h after injection of vehicle or Lat (n=8 per group). Statistical analysis: Quantification of F-actin (p=0.0060, t=3.232, *df*=14); G-actin (p=0.1425, t=1.554, *df*=14). Statistical comparison is performed using unpaired two-tailed t-test. Data are presented as mean±s.e.m. Statistical significance indicated as *p< 0.05, **p<0.01. Download Figure 2-1, TIF file

### Loss of dendritic spines in primary neurons from APP/PS1 mice occurs due to breakdown of F-actin nanoarchitecture

AD has been shown to be associated with changes in spine morphology and decreased synapse number (Blanpied and Ehlers, 2004); however, these changes have not been examined in detail within the synaptic compartments. To address this question, we used primary cortical neurons [day *in vitro* (DIV) 10 and16] from WT and APP/PS1 mice stained with DiI or phalloidin and captured images of at least two bits of tertiary neurites from each neuron before automated analysis using Neurolucida or MetaMorph, as appropriate. At DIV 10, there was no difference in the total number of spines, spine total extent, or spine surface area and only spine head diameter was decreased in APP/PS1 neurons compared with WT neurons (Fig. 3*A–C*). However, a significant reduction of F-actin (measured as phalloidin staining) was seen in APP/PS1 neurites (Fig. 3*D*,*E*), indicating that the F-actin decrease occurs very early, before spine maturation at DIV 10. When we examined neurons at DIV 16, we found that total dendritic spines, spine total extent, spine surface area, diameter of the spine head, and spine cross-sectional area (Fig. 3*F–H*) were all significantly decreased in APP/PS1 neurons. Furthermore, F-actin staining of dendritic spines using phalloidin showed a significant reduction in F-actin levels in tertiary neurites (Fig. 3*I*,*J*).

**Figure 4.**
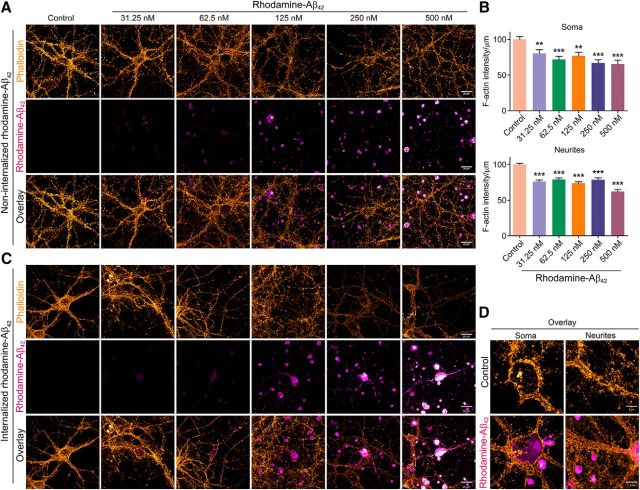
Exposure of primary cortical neurons to synthetic Aβ_42_ leads to decreased F-actin levels. ***A***, Primary cortical neurons (DIV 16) were treated with or without different doses of synthetic rhodamine-tagged Aβ_42_ (31.25, 62.5, 125, 250, or 500 nm) for 24 h. Neurons were then fixed and stained with phalloidin. Representative confocal images in pseudocolor are shown with rhodamine-Aβ_42_ in magenta-hot and phalloidin in orange hot (Alexa Fluor 488). Scale bar, 20 μm. Images shown are from one of three independent experiments. ***B***, Quantification of phalloidin signal from soma (*p* < 0.0001, *F* = 8.016, df = 142) and neurites (*p* < 0.0001, *F* = 32.7, df = 669). One-way ANOVA was used. *n* = 22–28 neurons from three independent experiments. Data are shown as mean ± SEM. Statistical significance: **p < 0.01,***p < 0.001. ***C***, Rhodamine-tagged Aβ_42_ is internalized by a small percentage (∼10–15%) of primary cortical neurons exposed to it. Representative confocal images show cells that have internalized rhodamine-tagged Aβ_42_. Intensity of phalloidin signal (orange hot) was too low to be quantified for several of the cases. Scale bar, 20 μm. ***D***, Representative high-resolution confocal images (magnified) are shown with rhodamine-Aβ_42_ in magenta hot and phalloidin in orange hot (Alexa Fluor 488). Imaging parameters were as follows: objective = 100×/1.4 NA, oil-immersion, pixel format = 1024 × 1024, bit depth = 16 bit, zoom factor = 1.8, and step size interval = 0.150 μm. Scale bar, 5 μm.

To determine whether depolymerization of F-actin was indeed caused by Aβ_42_ per se, we exposed primary cortical neurons to varying concentrations of rhodamine-tagged Aβ_42_ (31.25–500 nm) for 24 h and measured F-actin levels as the intensity of phalloidin labeling (Fig. 4*A–C*). Even at the lowest concentration examined, 31.25 nm, we saw a significant reduction in F-actin staining. The loss of F-actin increased with higher concentrations of rhodamine tagged Aβ_42_. Interestingly, a small fraction (∼10–15%) of neurons treated with rhodamine-Aβ_42_ internalized it, showing rhodamine signal corresponding to morphology of the neurons (Fig. 4*C*). The neurons that did internalize rhodamine-Aβ_42_ exhibited dramatic loss of phalloidin signal, even at lower concentrations (62.5 nm). Some of these neurons also appeared to have structural damage (such as discontinuous neurites and broken or blebbed membranes). The phalloidin signal for some of these neurons was completely lost and it was not possible to quantify the signal reliably. Therefore, we did observe differences in the depolymerization of F-actin *in vivo* in mice and in primary neurons. F-actin was lost from both soma and neurites in primary neurons. However, *in vivo*, in mice, we observed the loss only in synaptosomes and not in PNS. This could be due to the contribution of F-actin from other cells such as glia.

**Figure 5.**
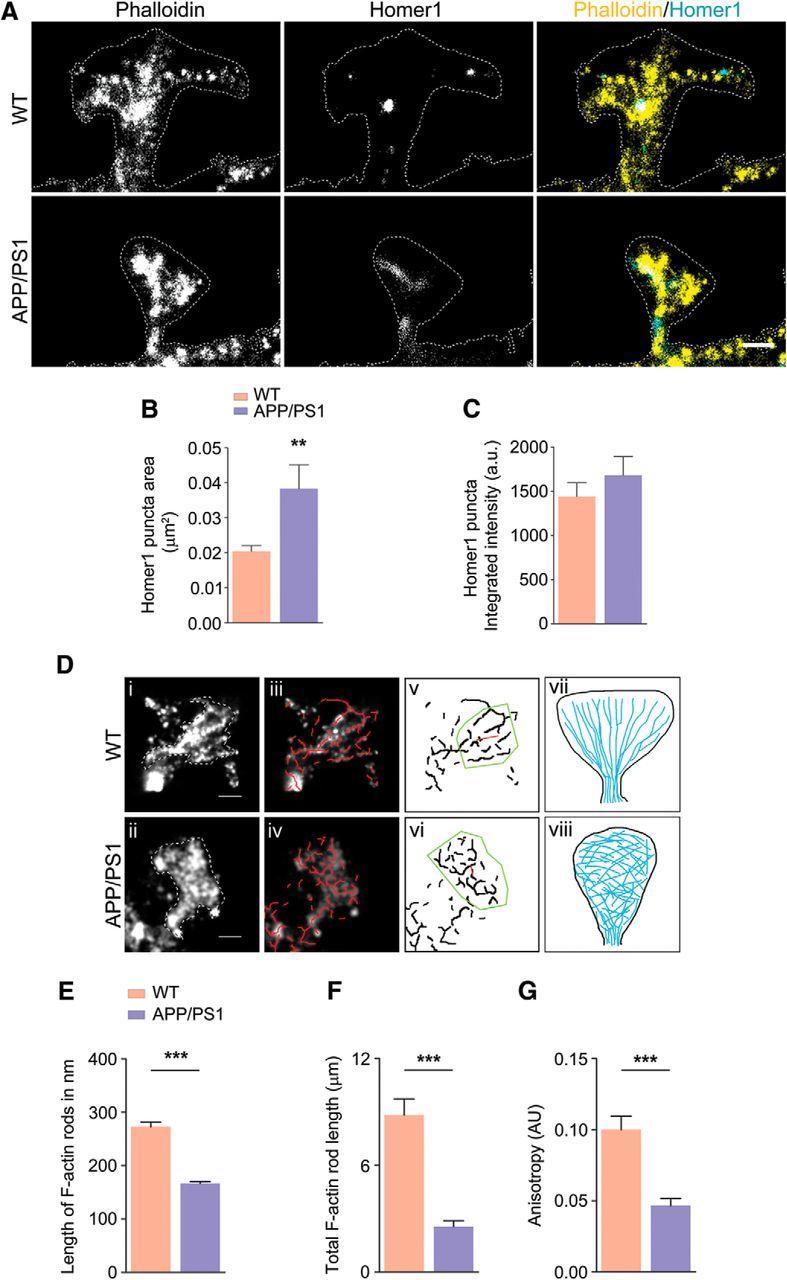
Perturbation of F-actin nanoarchitecture in dendritic spines of WT and APP/PS1 primary cortical neurons (see Fig. 5-1, and Fig. 5-2). ***A***, Representative reconstructed images of mushroom spines from WT and APP/PS1 primary neurons; merged images show phalloidin signal in yellow and Homer1 signal in cyan. ***B***, Homer1 puncta area showing a significant increase in case of APP/PS1 (*p* = 0.0014, *t* = 3.291, df = 102). ***C***, No change was detected in integrated intensity of Homer1 puncta (*p* = 0.3634, *t* = 0.9131, df = 102). ***D***, Automated filament detection and orientation were performed on representative WT (*i*) and APP/PS1 (*ii*) spines obtained using dSTORM imaging (*iii*) and indicate the overlay of automatically detected actin rods (red; *iv*) labeled with phalloidin–Alexa Fluor 647 in mushroom spines indicated in *i* and *ii*; *v* and *vi* indicate anisotropy of the actin organization within WT and APP/PS1, respectively. The length of the red line in the ROI (yellow) indicates the extent of anisotropy for the spines in *i* and *ii*, 0.19 for WT and 0.06 for APP/PS1, whereas the angle that it makes indicates average orientation of fibrils *vii* and *viii* indicate the scheme of actin distribution in WT, where actin rods are organized laterally, and in APP/PS1 spines, where this organization is lost. ***E***, Comparison of mean length of actin rods in WT and APP/PS1 spines indicating that WT spines have significantly longer actin structures within them compared with APP/PS1 spines. *n* = 30 for WT and *n* = 30 for APP/PS1 (*p* < 0.0001, *t* = 8.103, df = 1244). ***F***, Comparison of cumulative rod length in WT and APP/PS1 indicating the significant reduction of F-actin. *n* = 30 for WT and *n* = 30 for APP/PS1 (*p* < 0.0001, *t* = 6.491, df = 58). ***G***, Comparison of mean anisotropy of WT and APP/PS1 spines indicating that WT spines (*n* = 25) have significantly more directed actin structures within them compared with APP/PS1 spines (*n* = 25) (*p* < 0. 0001, *t* = 5.015, df = 49). Statistical comparison was performed using unpaired two-tailed *t* test. **p < 0.01,***p < 0.001. Scale bar, 500 nm.

We then performed dSTORM (Rust et al., 2006; van de Linde et al., 2011; Tang et al., 2016) of F-actin at mushroom spine heads (as indicated by morphology and presence of Homer1 puncta) in WT neurons and APP/PS1 neurons actin is arranged as radiating structures from the base of the spine head (Fig. 5*A*). On performing automated analysis on randomly selected mushroom spines, it was observed that this arrangement was well conserved in mushroom spines from WT neurons. Using automated detection of radiating actin rods >100 nm, it was observed that the mean rod length was 272.9 ± 8.925 nm in WT and 166.7 ± 3.418 nm in APP/PS1 primary neurons (Fig. 5*E*). We then calculated the distribution of the number of actin rods in mushroom spines of both WT and APP/PS1 primary neurons. In the next step, we calculated the cumulative rod length per spine and found that the mean cumulative rod length for the WT spine was 8.827 ± 0.9043 μm and for the APP/PS1 spine, it was 2.556 ± 0.3403 μm. To understand whether the lateral organization of the radiating rods was disturbed, we calculated the anisotropy, indicating the lateral spread of the radiating actin rods using Fibriltool. A significant change in the distribution of anisotropy of the actin rods was observed. The WT neurons showed an anisotropy of 0.1001 ± 0.009512, whereas it was 0.04665 ± 0.005050 for APP/PS1 primary neurons (Fig. 5-2*A*,*B*). The significant reduction in the length and anisotropy of actin rods indicate significant alteration of the F-actin nanoarchitecture in mushroom spines from APP/PS1 primary neurons.

10.1523/JNEUROSCI.2127-17.2017.f5-1 Figure 5-1 Determination of the spatial resolution of dSTORM (Refer to Figure 5 in the main article) (A) point-spread function of a single fluorophore, (B) localization pattern of a fluorescent bead that was localized 4000 times (C) histogram of the standard deviation of localizations of 60 single-molecule point-spread functions (average standard deviation 19.4 nm) and (D) histogram of the full-width half-maximum (FWHM) of 60 single-molecule point-spread functions (average FWHM 44.6 nm). Download Figure 5-1, TIF file

10.1523/JNEUROSCI.2127-17.2017.f5-2 Figure 5-2 dSTORM image analysis (Refer to Figure 5 in the main article). An illustration of analysis on multiple spines in both WT a), b), c), and APP/PS1 j), k), l). d), e), f), m), n), and o) using Ridge Detection plugin rods (red) are identified. g), h), i), p), q) and r) are binary images obtained after rod analysis from d), e), f), m), n), and o) were subjected to anisotropy measurement in the spine head using FibrilTool plugin. The length of the red line indicates the extent of anisotropy which for WT are g) 0.1613; h) 0.0912; i) 0.1321 and APP/PS1 are p) 0.0534 , q) 0.0246 and r) 0.0538. Scale bar=500 nm. Download Figure 5-2, TIF file

### Hyperactivation of ADF/cofilin caused by decreased cofilin phosphorylation levels contribute to F-actin loss

**Figure 6.**
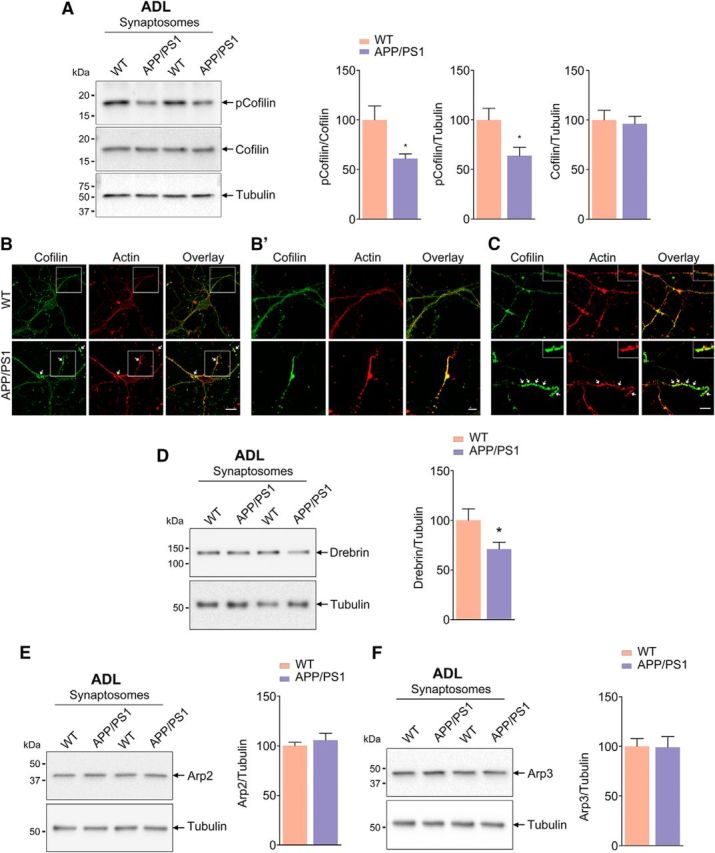
Decreased ADF/cofilin phosphorylation and Drebrin levels in synaptosomes of APP/PS1 mice (see Fig. 6-1). ***A***, Phosphorylation of cofilin (Ser3) is reduced in synaptosomes isolated from ADL APP/PS1 mice compared with age-matched WT mice. Statistical analysis: p-cofilin/cofilin, *p* = 0.0181, *t* = 2.631, df = 16; p-cofilin/tubulin, *p* = 0.0238, *t* = 2.498, df = 16; cofilin/tubulin, *p* = 0.7692, *t* = 0.3051, df = 16; *n* = 8–9 per group. ***B***, ***C***, ADF/cofilin-actin rods observed in primary neurons derived from APP/PS1 mice. Representative confocal images of primary cortical neurons from WT and APP/PS1 mice immunostained for actin (red) and cofilin (green). Arrows indicates ADF/cofilin-actin rods (***B′***). Insets show magnified images. Scale bar, 5 μm. ***D***, Drebrin levels are reduced in synaptosomes isolated from ADL APP/PS1 mice compared with age-matched WT mice. Statistical analysis: Drebrin/tubulin, *p* = 0.0458, *t* = 2.146, df = 18; *n* = 10 per group. ***E***, ***F***, Arp2 and Arp3 protein levels are unaffected in synaptosomes isolated from ADL APP/PS1 mice compared with age-matched WT mice. Statistical analysis: Arp2/tubulin, *p* = 0.4899, *t* = 0.7090, df = 14; Arp3/tubulin, *p* = 0.9445, *t* = 0.07088, df = 14; *n* = 8 per group. Unpaired two-tailed *t* test was used. Asterisks indicate statistical significance for WT versus APP/PS1.

ADF/cofilin and drebrin are some of the major regulators of actin polymerization (Bamburg, 1999; Bamburg and Bernstein, 2016). Dephosphorylation of p-cofilin leads to active cofilin, which binds to actin and promotes conversion of F-actin to G-actin (Bamburg and Bernstein, 2016). The inactive p-cofilin and p-cofilin/cofilin levels were significantly decreased in synaptosomes prepared from ADL (1-month-old) APP/PS1 cortex, although ADF/cofilin levels per se were unaltered (Fig. 6*A*). This indicates that the lowered p-cofilin to cofilin ratio would promote F-actin depolymerization at the synapse as seen in Figure 6, *A* and *C*. When we examined the above in MA (9-month-old) animals, we found that the p-cofilin to ADF/cofilin ratio was also dramatically reduced in synaptosomes, indicating disruption of F-actin homeostasis (Fig. 6-1*A*). In addition, coimmunostaining of ADF/cofilin and actin in primary cortical neurons from APP/PS1 mice showed ADF/cofilin-actin rods within neurites under physiological conditions (Fig. 6*B*), but not in WT neurons. These results indicate that ADF/cofilin-actin rod formation led to synaptic dysfunctions in APP/PS1 mice by sequestering ADF/cofilin and disrupting actin dynamics. In addition, drebrin, which positively regulates polymerization of actin to F-actin was also decreased in the synaptosomes from ADL APP/PS1 mice (Fig. 6*D*). Interestingly, Arp2/3, which promotes actin polymerization, was unaffected (Fig. 6*E*,*F*), indicating that positive regulators of actin polymerization are not affected globally but rather selectively. We have therefore demonstrated that a decrease of drebrin and p-cofilin could potentially result in dysregulation of F-/G-actin homeostasis in synaptosomes.

10.1523/JNEUROSCI.2127-17.2017.f6-1 Figure 6-1 ADF/cofilin phosphorylation and Drebrin levels are decreased in synaptosomes of middle aged (MA) APP/PS1 mice (Refer to Figure 6 in the main article). (A) Phosphorylation of cofilin (Ser3) is decreased in synaptosomes isolated from middle aged (MA) APP/PS1 mice compared to age-matched WT mice. Statistical analysis: p-cofilin/cofilin (p=0.0334, t=2.359, *df*=14); p-cofilin/tubulin (p=0.0363, t=2.315, *df*=14); Cofilin/tubulin (p=0.4515, t=0.7746, *df*=14). (n=8 per group). (B) Drebrin levels are decreased in synaptosomes isolated from middle aged (MA) APP/PS1 mice compared to age-matched WT mice. Statistical analysis: Drebrin/tubulin (p=0.0277, t=2.457, *df*=14). (n=8 per group). (C-D) Arp2 and Arp3 protein levels are unaffected in synaptosomes isolated from middle aged (MA) APP/PS1 mice compared to age-matched WT mice. Statistical analysis: Arp2/tubulin (p=0.8067, t=0.2512, *df*=10); Arp3/tubulin (p=0.3233, t=1.039, *df*=10). (n=6 per group). Unpaired two-tailed t-test.* indicates statistical significance for wild type versus APP/PS1. Download Figure 6-1, TIF file

### Loss of synaptosomal F-actin levels in cortex of older persons

**Figure 7.**
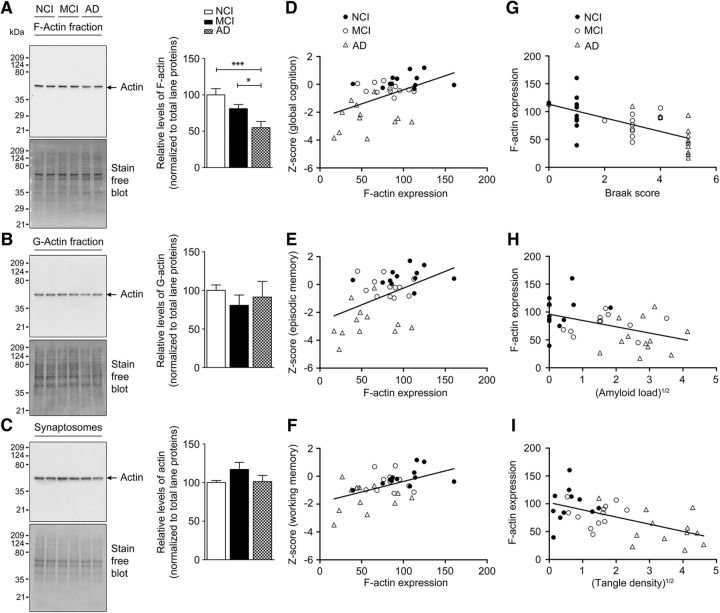
Decreased F-actin levels in synaptosomes from AD patients. ***A***–***C***, Enriched F-actin and G-actin fractions were isolated from synaptosomes of NCI, MCI, and AD human brain cortex samples. All fractions were subjected to TGX-stain-free gels and immunoblotted with antibody to actin. Densitometric analyses for F-actin, G-actin, and total actin levels were normalized to total lane proteins. Results are shown as the mean ± SEM of 12 samples per group except in the case of synaptosomes, in which *n* = 8 per group. Statistical analysis: ***A***, quantification of F-actin, *p* = 0.0011, *F* = 8.475, df = 35; ***B***, quantification of G-actin, *p* = 0.6493, *F* = 0.4375, df = 35; ***C***, quantification of total actin (*p* = 0.1937, *F* = 1.777, df = 23). One-way ANOVA followed by Newman–Keuls post test was used. *p < 0.05, ***p < 0.001 indicates statistical significance for control versus AD and MCI versus AD (*n* = 36). F-actin loss is associated with loss of memory-related cognitive performance and AD-related histopathology. Selected GLM analyses results from Tables 1 and 2 have been plotted for the primary factors. ***D***–***F***, Effects of F-actin expression on cognitive performance after controlling for education and age at death with regression curves plotting global cognitive score (***D***), episodic memory score (***E***), and working memory score (***F***) against synaptosomal F-actin. ***G***–***I***, Effects of AD-related histopathological scores on F-actin expression after controlling for postmortem delay and age at death with regression curves plotting synaptosomal F-actin levels against Braak scores (***G***), square root of amyloid load (***H***), and square root of tangle density (***I***). Respective *p*-values are listed in Tables 1 and 2 (*n* = 36).

To determine whether the loss of synaptosomal F-actin seen in model systems (primary neurons and APP/PS1 mice) extrapolates to human subjects with AD, we assayed F-actin levels in synaptosomes prepared from postmortem tissue of subjects with NCI, MCI, and AD. Quantitative immunoblot analysis showed loss of synaptosomal F-actin, but not G-actin or total actin (Fig. 7*A–C*). A significant correlation was also seen between the loss of synaptosomal F-actin, but not G-actin (all *p* > 0.28), and performance on global cognition, which was driven by associations with episodic memory and working memory, but not semantic memory, perceptual speed, or perceptual orientation (Fig. 7*D–F*, Table 1). Synaptosomal F-actin levels, but not G-actin (all *p* > 0.10), were also associated with in Braak staging, β-amyloid load, and tangle density (Fig. 7*G–I*, Table 2).

**Table 1.** Change in F-actin expression levels are associated with altered cognitive performance, especially in the memory domain (see Fig. 7*D*–*F*)

Dependent variable	*p*-value	Nature of association
Cogn_Glob	**0.0029962**	Positive
Cogn_Epi	**0.0032389**	Positive
Cogn_WM	**0.0032874**	Positive
Cogn_Sem	0.13209	Nonsignificant
Cogn_PS	0.2242	Nonsignificant
Cogn_PO	0.24496	Nonsignificant

The predictor variable was F-actin and the control predictor variables were education and age at death. Ordinary least-squares regression-based GLM analysis (without considering variable interactions) demonstrates the effects of F-actin expression levels on different cognitive scores when controlled for education and age at death.

*n* = 36 (*n* = 12 from each group). Bold font indicates significant *p*-values.

**Table 2.** Increased AD-related histopathology is associated with decrease in F-actin expression levels (see Fig. 7*G*–*I*)

Predictor variable1	*p*-value	Nature of association
Braak staging	5.60E-05	Negative
Square root amyloid load	0.011846	Negative
Square root tangle density	0.00062438	Negative

Control predictor variables were PMI and age at death. The dependent variable was F-actin. Ordinary least-squares regression-based GLM analysis (without considering variable interactions) demonstrates the effects of different AD-related histopathological scores such as Braak staging, total amyloid load, and total tangle density on the expression of F-actin when controlled for postmortem interval (PMI) and age at death.

*n* = 36 (*n* = 12 from each group).

## Discussion

Synaptic dysfunction, as shown by decreased utilization of FDG on PET imaging in humans, occurs decades before the onset of symptoms, indicating that synaptic function is deregulated early in the pathogenesis of AD (Jack and Holtzman, 2013; Jack et al., 2013). In primary neurons from AD mice, loss of spines is also seen early in the disease progression (Herms and Dorostkar, 2016). In addition, transient LTP is reduced in APP/PS1 mice at 3 months of age (Volianskis et al., 2010). However, the molecular underpinnings of these phenomena are unclear. F-actin is the major cytoskeletal protein in spines and actin treadmilling (i.e., dynamics of F-actin/G-actin ratio) is critical for structural changes in spines that typically occur during synaptic activity, including memory formation (Hotulainen and Hoogenraad, 2010; Huang et al., 2013). Here, we demonstrate that F-actin in spines is disassembled, leading to alteration of the cytoskeletal architecture of the spines in primary neurons derived from APP/PS1 mice and in synaptosomes from 1- and 9-month-old APP/PS1 mouse cortex and human cortical tissue from MCI and AD subjects. The loss of F-actin due to depolymerization (because total actin levels are unchanged) is seen only in synaptosomes and not in the postnuclear supernatant, indicating the specificity and selectivity of the effect.

Importantly, we could measure deficits in memory reliably using cFC in 2-month-old APP/PS1 mice. This is the earliest recorded behavioral deficit; earlier reports have shown impaired LTP at 3 months and APP/PS1 mice show cognitive deficits in the contextual memory and its extinction at 4–6 months of age (Kilgore et al., 2010; Bonardi et al., 2011). Because F-actin is known to play a critical role in memory formation (Okamoto et al., 2004), we administered jasplakinolide, a molecule that stabilizes actin filaments, intrathecally to APP/PS1 mice and found that the cFC deficit could be completely reversed (along with restoration of synaptosomal F-actin levels), indicating that F-actin deficiency indeed contributes to the cFC deficit seen in APP/PS1 mice. Conversely, latrunculin A, an actin-depolymerizing agent, was able to induce cFC deficit in WT mice when given intrathecally, indicating clearly the critical role of synaptosomal F-actin in consolidation of memory. Further, the decrease of F-actin was also seen in synaptosomes prepared from cortex obtained postmortem from human subjects with AD and MCI compared with normal controls and this decrease showed significant correlation with impaired cognitive performance. The decrease in synaptosomal F-actin also correlated inversely with pathological scores such as β-amyloid and tangle load and Braak staging. Therefore, our results indicate that loss of F-actin at the synapse is a critical event that occurs early in the disease process in mouse models and humans.

F-actin has specialized organization in spines when seen using dSTORM, as depicted in Figure 5*A*, wherein the F-actin rods radiate outwards from the base to the spine head. This organization of F-actin is altered in neurons from APP/PS1 mice. Interestingly, the postsynaptic density scaffolding protein Homer1 is dispersed sparsely across the spine in neurons from APP/PS1 mice, whereas they are seen as distinct puncta covering a smaller area in neurons from WT mice. These images at high resolution indicate the partial breakdown of the cytoskeletal architecture maintained by F-actin in APP/PS1 neurons.

We performed experiments wherein Aβ_42_ was added externally (31.25 nm onwards) for 24 h and found that it leads to substantial loss of F-actin. The few neurons that did internalize Aβ_42_ showed no detectable phalloidin staining, which could be due to leaky membranes of dying neurons. Therefore, Aβ_42_ exposure leads to F-actin loss regardless of whether it is added exogenously to neurons or generated endogenously. Recent reports have suggested that pathogenic events seen in APP/PS1 mice could be due to enhanced expression of APP rather than being a consequence of increased Aβ_42_ (Saito et al., 2016). The experiments showing F-actin loss in primary neurons exposed to low levels of Aβ_42_ demonstrate that F-actin loss is indeed due to increased Aβ_42_.

The ADF/cofilin family members drebrin and Arp2/3 are some of the most important regulators of actin polymerization. ADF/cofilin binds to F-actin in its nonphosphorylated active form and promotes its depolymerization (Hotulainen et al., 2005; Bamburg and Bernstein, 2010). It promotes actin assembly or disassembly depending on the concentrations of cofilin relative to actin and other actin-interacting proteins. It is well established that, if the ratio of cofilin/actin subunits in a filament is at physiological range (<1%) (Bernstein and Bamburg, 2010), then F-actin is severed, generating more monomers and resulting in increased treadmilling of actin with the utilization of ATP. At much higher cofilin/actin ratio (1:10 to 1:2), cofilin breaks F-actin speedily. However, this effect is fleeting because it binds ADP actin and stabilizes it in a twisted form (rods), thus stalling F-actin synthesis. The formation of ADF/cofilin-actin rods has been considered as an energy-conserving mechanism and can be reversed. However, the formation of such rods within neurites interferes with transport along neurites to synaptic terminals, potentially leading to synapse loss. It remains to be elucidated whether and exactly how the relative concentrations of cofilin to actin contribute to the spatial coordination of actin dynamics that underlies normal synaptic function. In addition to the above, another important determinant of F-actin destabilization is the availability of ATP. Because mitochondrial dysfunction leading to ATP loss has been reported in brains of AD mouse models and in cells exposed to Aβ_42_ (Du et al., 2012), it is plausible that an inadequate ATP concentration could also contribute to impaired treadmilling of actin.

Although ADF/cofilin-actin rods have been described in neurons exposed to Aβ_42_ (600 nm) or hydrogen peroxide (Bamburg and Bernstein, 2016), we demonstrate the formation of these rods in primary neurons from APP/PS1 mice. Phosphorylation of cofilin is decreased in synaptosomes from APP/PS1 mice starting at 1 month of age and is sustained even at 9 months of age, indicating the disruption of the p-cofilin/cofilin ratio through the observed lifespan. Whereas defects in p-cofilin/cofilin ratio have been studied before using brain lysates, the results have been contradictory (Barone et al., 2014). Although some reports have shown decreased p-cofilin levels (Woo et al., 2015), others have shown increased p-cofilin (Heredia et al., 2006; Barone et al., 2014). We now report the significant and sustained decrease in p-cofilin levels in synaptosomes, indicating that this may have a direct effect on depolymerization of F-actin. In fact, ADF/cofilin-actin rods, described earlier as being indicators of F-actin destabilization, are seen abundantly in APP/PS1 neurons (Fig. 6*B*). Although the upstream regulators of ADF/cofilin such as RAC, CDC42, PAK (Zhao et al., 2006), and LIMK have been reported to be diminished in AD brain and mouse models of AD (Barone et al., 2014), the consequence of this dysregulation on ADF/cofilin and the resulting F-actin destabilization (and altered treadmilling of actin) has not been investigated. We know that overexpression of PAK attenuates AD pathology, but whether this is through F-actin is unclear. Drebrin, an actin-regulatory protein, is also decreased in hippocampal lysates from AD patients (Zhao et al., 2006; Counts et al., 2012) and in a mouse model (Liu et al., 2015); however, the consequence of this on F-actin in dendritic spines is unclear. In this study, we demonstrate that F-actin nanoarchitecture is disassembled (as seen by dSTORM of F-actin) in spines due to loss of F-actin (as seen biochemically) in synaptosomes of AD mice even as early 1 month of age, indicating that synaptic dysfunction starts early in the disease process. In APP/PS1 mice, although the brain Aβ_42_ levels are quite low, we see significant amounts of soluble Aβ oligomers in synaptosomes from 1-month-old APP/PS1 mice, indicating that their presence at the synapse could potentially initiate pathogenic events (Ahmad et al., 2017).

The loss of synaptosomal F-actin seen in a mouse model was replicated in synaptosomes prepared from human postmortem cortical tissue from persons with NCI, MCI, and AD, in which a graded lowering of synaptosomal F-actin was seen. We observed a significant positive correlation between synaptosomal F-actin loss and measures of global cognition due to strong associations with episodic and working memory. Finally, there was a negative correlation with Braak stage, β-amyloid, and tangle density. These results indicate that synaptosomal F-actin, which is crucial for memory consolidation, is impaired in human subjects during their pathological trajectory toward AD and point to the importance of this finding in terms of understanding early synaptic dysfunction in AD. It remains to be determined whether similar perturbation of F-/G-actin homeostasis occurs in other neurodegenerative diseases.

## References

[B1] Ahmad F, Singh K, Das D, Gowaikar R, Shaw E, Ramachandran A, Rupanagudi KV, Kommaddi RP, Bennett DA, Ravindranath V (2017) Reactive oxygen species-mediated loss of synaptic Akt1 signaling leads to deficient activity-dependent protein translation early in Alzheimer's disease. Antioxid Redox Signal 27:1269–1280. 10.1089/ars.2016.6860 28264587 PMC5655421

[B2] Bamburg JR (1999) Proteins of the ADF/cofilin family: essential regulators of actin dynamics. Annu Rev Cell Dev Biol 15:185–230. 10.1146/annurev.cellbio.15.1.185 10611961

[B3] Bamburg JR, Bernstein BW (2010) Roles of ADF/cofilin in actin polymerization and beyond. F1000 Biol Rep 2:62. 10.3410/B2-62 21173851 PMC2990448

[B4] Bamburg JR, Bernstein BW (2016) Actin dynamics and cofilin-actin rods in alzheimer disease. Cytoskeleton (Hoboken) 73:477–497. 10.1002/cm.21282 26873625 PMC5345344

[B5] Bamburg JR, Bernstein BW, Davis RC, Flynn KC, Goldsbury C, Jensen JR, Maloney MT, Marsden IT, Minamide LS, Pak CW, Shaw AE, Whiteman I, Wiggan O (2010) ADF/Cofilin-actin rods in neurodegenerative diseases. Curr Alzheimer Res 7:241–250. 10.2174/156720510791050902 20088812 PMC4458070

[B6] Barone E, Mosser S, Fraering PC (2014) Inactivation of brain Cofilin-1 by age, Alzheimer's disease and gamma-secretase. Biochim Biophys Acta 1842:2500–2509. 10.1016/j.bbadis.2014.10.004 25315299

[B7] Beaudoin GM 3rd, Lee SH, Singh D, Yuan Y, Ng YG, Reichardt LF, Arikkath J (2012) Culturing pyramidal neurons from the early postnatal mouse hippocampus and cortex. Nat Protoc 7:1741–1754. 10.1038/nprot.2012.099 22936216

[B8] Bennett DA, Wilson RS, Schneider JA, Evans DA, Beckett LA, Aggarwal NT, Barnes LL, Fox JH, Bach J (2002) Natural history of mild cognitive impairment in older persons. Neurology 59:198–205. 10.1212/WNL.59.2.198 12136057

[B9] Bennett DA, Schneider JA, Wilson RS, Bienias JL, Arnold SE (2004) Neurofibrillary tangles mediate the association of amyloid load with clinical Alzheimer disease and level of cognitive function. Arch Neurol 61:378–384. 10.1001/archneur.61.3.378 15023815

[B10] Bennett DA, Schneider JA, Bienias JL, Evans DA, Wilson RS (2005) Mild cognitive impairment is related to Alzheimer disease pathology and cerebral infarctions. Neurology 64:834–841. 10.1212/01.WNL.0000152982.47274.9E 15753419

[B11] Bennett DA, Schneider JA, Aggarwal NT, Arvanitakis Z, Shah RC, Kelly JF, Fox JH, Cochran EJ, Arends D, Treinkman AD, Wilson RS (2006) Decision rules guiding the clinical diagnosis of Alzheimer's disease in two community-based cohort studies compared to standard practice in a clinic-based cohort study. Neuroepidemiology 27:169–176. 10.1159/000096129 17035694

[B12] Bennett DA, Schneider JA, Arvanitakis Z, Wilson RS (2012) Overview and findings from the religious orders study. Curr Alzheimer Res 9:628–645. 10.2174/156720512801322573 22471860 PMC3409291

[B13] Bernstein BW, Bamburg JR (2010) ADF/cofilin: a functional node in cell biology. Trends Cell Biol 20:187–195. 10.1016/j.tcb.2010.01.001 20133134 PMC2849908

[B14] Blanpied TA, Ehlers MD (2004) Microanatomy of dendritic spines: emerging principles of synaptic pathology in psychiatric and neurological disease. Biol Psychiatry 55:1121–1127. 10.1016/j.biopsych.2003.10.006 15184030

[B15] Bonardi C, de Pulford F, Jennings D, Pardon MC (2011) A detailed analysis of the early context extinction deficits seen in APPswe/PS1dE9 female mice and their relevance to preclinical Alzheimer's disease. Behav Brain Res 222:89–97. 10.1016/j.bbr.2011.03.041 21440575

[B16] Bosch M, Castro J, Saneyoshi T, Matsuno H, Sur M, Hayashi Y (2014) Structural and molecular remodeling of dendritic spine substructures during long-term potentiation. Neuron 82:444–459. 10.1016/j.neuron.2014.03.021 24742465 PMC4281348

[B17] Boudaoud A, Burian A, Borowska-Wykręt D, Uyttewaal M, Wrzalik R, Kwiatkowska D, Hamant O (2014) FibrilTool, an ImageJ plug-in to quantify fibrillar structures in raw microscopy images. Nat Protoc 9:457–463. 10.1038/nprot.2014.024 24481272

[B18] Chazeau A, Mehidi A, Nair D, Gautier JJ, Leduc C, Chamma I, Kage F, Kechkar A, Thoumine O, Rottner K, Choquet D, Gautreau A, Sibarita JB, Giannone G (2014) Nanoscale segregation of actin nucleation and elongation factors determines dendritic spine protrusion. EMBO J 33:2745–2764. 10.15252/embj.201488837 25293574 PMC4282554

[B19] Colella AD, Chegenii N, Tea MN, Gibbins IL, Williams KA, Chataway TK (2012) Comparison of Stain-Free gels with traditional immunoblot loading control methodology. Anal Biochem 430:108–110. 10.1016/j.ab.2012.08.015 22929699

[B20] Counts SE, He B, Nadeem M, Wuu J, Scheff SW, Mufson EJ (2012) Hippocampal drebrin loss in mild cognitive impairment. Neurodegener Dis 10:216–219. 10.1159/000333122 22310934 PMC3363353

[B21] Cummings DM, Liu W, Portelius E, Bayram S, Yasvoina M, Ho SH, Smits H, Ali SS, Steinberg R, Pegasiou CM, James OT, Matarin M, Richardson JC, Zetterberg H, Blennow K, Hardy JA, Salih DA, Edwards FA (2015) First effects of rising amyloid-beta in transgenic mouse brain: synaptic transmission and gene expression. Brain 138:1992–2004. 10.1093/brain/awv127 25981962 PMC4572488

[B22] Dickstein DL, Dickstein DR, Janssen WG, Hof PR, Glaser JR, Rodriguez A, O'Connor N, Angstman P, Tappan SJ (2016) Automatic dendritic spine quantification from confocal data with Neurolucida 360. Curr Protoc Neurosci 77:1.27.1–1.27.21. 10.1002/cpns.16 27696360 PMC5113738

[B23] Dillon C, Goda Y (2005) The actin cytoskeleton: integrating form and function at the synapse. Annu Rev Neurosci 28:25–55. 10.1146/annurev.neuro.28.061604.135757 16029114

[B24] Du H, Guo L, Yan SS (2012) Synaptic mitochondrial pathology in Alzheimer's disease. Antioxid Redox Signal 16:1467–1475. 10.1089/ars.2011.4277 21942330 PMC3329948

[B25] Efimova N, Korobova F, Stankewich MC, Moberly AH, Stolz DB, Wang J, Kashina A, Ma M, Svitkina T (2017) BetaIII spectrin is necessary for formation of the constricted neck of dendritic spines and regulation of synaptic activity in neurons. J Neurosci 37:6442–6459. 10.1523/JNEUROSCI.3520-16.2017 28576936 PMC5511878

[B26] Franklin KBJ, Paxinos G (1997) The mouse brain in stereotaxic coordinates. San Diego, CA: Academic Press.

[B27] Frost NA, Shroff H, Kong H, Betzig E, Blanpied TA (2010) Single-molecule discrimination of discrete perisynaptic and distributed sites of actin filament assembly within dendritic spines. Neuron 67:86–99. 10.1016/j.neuron.2010.05.026 20624594 PMC2904347

[B28] Gilda JE, Gomes AV (2013) Stain-Free total protein staining is a superior loading control to beta-actin for Western blots. Anal Biochem 440:186–188. 10.1016/j.ab.2013.05.027 23747530 PMC3809032

[B29] Gilda JE, Gomes AV (2015) Western blotting using in-gel protein labeling as a normalization control: stain-free technology. Methods Mol Biol 1295:381–391. 10.1007/978-1-4939-2550-6_27 25820735

[B30] Gu J, Lee CW, Fan Y, Komlos D, Tang X, Sun C, Yu K, Hartzell HC, Chen G, Bamburg JR, Zheng JQ (2010) ADF/cofilin-mediated actin dynamics regulate AMPA receptor trafficking during synaptic plasticity. Nat Neurosci 13:1208–1215. 10.1038/nn.2634 20835250 PMC2947576

[B31] Gürtler A, Kunz N, Gomolka M, Hornhardt S, Friedl AA, McDonald K, Kohn JE, Posch A (2013) Stain-Free technology as a normalization tool in Western blot analysis. Anal Biochem 433:105–111. 10.1016/j.ab.2012.10.010 23085117

[B32] Heredia L, Helguera P, de Olmos S, Kedikian G, Solá Vigo F, LaFerla F, Staufenbiel M, de Olmos J, Busciglio J, Cáceres A, Lorenzo A (2006) Phosphorylation of actin-depolymerizing factor/cofilin by LIM-kinase mediates amyloid beta-induced degeneration: a potential mechanism of neuronal dystrophy in Alzheimer's disease. J Neurosci 26:6533–6542. 10.1523/JNEUROSCI.5567-05.2006 16775141 PMC6674046

[B33] Herms J, Dorostkar MM (2016) Dendritic spine pathology in neurodegenerative diseases. Annu Rev Pathol 11:221–250. 10.1146/annurev-pathol-012615-044216 26907528

[B34] Holcomb L, Gordon MN, McGowan E, Yu X, Benkovic S, Jantzen P, Wright K, Saad I, Mueller R, Morgan D, Sanders S, Zehr C, O'Campo K, Hardy J, Prada CM, Eckman C, Younkin S, Hsiao K, Duff K (1998) Accelerated Alzheimer-type phenotype in transgenic mice carrying both mutant amyloid precursor protein and presenilin 1 transgenes. Nat Med 4:97–100. 10.1038/nm0198-097 9427614

[B35] Hotulainen P, Hoogenraad CC (2010) Actin in dendritic spines: connecting dynamics to function. J Cell Biol 189:619–629. 10.1083/jcb.201003008 20457765 PMC2872912

[B36] Hotulainen P, Paunola E, Vartiainen MK, Lappalainen P (2005) Actin-depolymerizing factor and cofilin-1 play overlapping roles in promoting rapid F-actin depolymerization in mammalian nonmuscle cells. Mol Biol Cell 16:649–664. 15548599 10.1091/mbc.E04-07-0555 PMC545901

[B37] Hotulainen P, Llano O, Smirnov S, Tanhuanpää K, Faix J, Rivera C, Lappalainen P (2009) Defining mechanisms of actin polymerization and depolymerization during dendritic spine morphogenesis. J Cell Biol 185:323–339. 10.1083/jcb.200809046 19380880 PMC2700375

[B38] Huang W, Zhu PJ, Zhang S, Zhou H, Stoica L, Galiano M, Krnjević K, Roman G, Costa-Mattioli M (2013) mTORC2 controls actin polymerization required for consolidation of long-term memory. Nat Neurosci 16:441–448. 10.1038/nn.3351 23455608 PMC3615448

[B39] Hung RJ, Spaeth CS, Yesilyurt HG, Terman JR (2013) SelR reverses Mical-mediated oxidation of actin to regulate F-actin dynamics. Nat Cell Biol 15:1445–1454. 10.1038/ncb2871 24212093 PMC4254815

[B40] Izeddin I, Boulanger J, Racine V, Specht CG, Kechkar A, Nair D, Triller A, Choquet D, Dahan M, Sibarita JB (2012) Wavelet analysis for single molecule localization microscopy. Opt Express 20:2081–2095. 10.1364/OE.20.002081 22330449

[B41] Jack CR Jr, Holtzman DM (2013) Biomarker modeling of Alzheimer's disease. Neuron 80:1347–1358. 10.1016/j.neuron.2013.12.003 24360540 PMC3928967

[B42] Jack CR Jr, Knopman DS, Jagust WJ, Petersen RC, Weiner MW, Aisen PS, Shaw LM, Vemuri P, Wiste HJ, Weigand SD, Lesnick TG, Pankratz VS, Donohue MC, Trojanowski JQ (2013) Tracking pathophysiological processes in Alzheimer's disease: an updated hypothetical model of dynamic biomarkers. Lancet Neurol 12:207–216. 10.1016/S1474-4422(12)70291-0 23332364 PMC3622225

[B43] Kilgore M, Miller CA, Fass DM, Hennig KM, Haggarty SJ, Sweatt JD, Rumbaugh G (2010) Inhibitors of class 1 histone deacetylases reverse contextual memory deficits in a mouse model of Alzheimer's disease. Neuropsychopharmacology 35:870–880. 10.1038/npp.2009.197 20010553 PMC3055373

[B44] Korobova F, Svitkina T (2010) Molecular architecture of synaptic actin cytoskeleton in hippocampal neurons reveals a mechanism of dendritic spine morphogenesis. Mol Biol Cell 21:165–176. 10.1091/mbc.E09-07-0596 19889835 PMC2801710

[B45] Lesné S, Koh MT, Kotilinek L, Kayed R, Glabe CG, Yang A, Gallagher M, Ashe KH (2006) A specific amyloid-beta protein assembly in the brain impairs memory. Nature 440:352–357. 10.1038/nature04533 16541076

[B46] Liu DS, Pan XD, Zhang J, Shen H, Collins NC, Cole AM, Koster KP, Ben Aissa M, Dai XM, Zhou M, Tai LM, Zhu YG, LaDu M, Chen XC (2015) APOE4 enhances age-dependent decline in cognitive function by down-regulating an NMDA receptor pathway in EFAD-Tg mice. Mol Neurodegener 10:7. 10.1186/s13024-015-0002-2 25871877 PMC4391134

[B47] Mendoza-Naranjo A, Contreras-Vallejos E, Henriquez DR, Otth C, Bamburg JR, Maccioni RB, Gonzalez-Billault C (2012) Fibrillar amyloid-beta1–42 modifies actin organization affecting the cofilin phosphorylation state: a role for Rac1/cdc42 effector proteins and the slingshot phosphatase. J Alzheimers Dis 29:63–77. 10.3233/JAD-2012-101575 22204905

[B48] Nair D, Hosy E, Petersen JD, Constals A, Giannone G, Choquet D, Sibarita JB (2013) Super-resolution imaging reveals that AMPA receptors inside synapses are dynamically organized in nanodomains regulated by PSD95. J Neurosci 33:13204–13224. 10.1523/JNEUROSCI.2381-12.2013 23926273 PMC6619720

[B49] Nanguneri S, Flottmann B, Horstmann H, Heilemann M, Kuner T (2012) Three-dimensional, tomographic super-resolution fluorescence imaging of serially sectioned thick samples. PLoS One 7:e38098. 10.1371/journal.pone.0038098 22662272 PMC3360663

[B50] Okamoto K, Nagai T, Miyawaki A, Hayashi Y (2004) Rapid and persistent modulation of actin dynamics regulates postsynaptic reorganization underlying bidirectional plasticity. Nat Neurosci 7:1104–1112. 10.1038/nn1311 15361876

[B51] Okamoto K, Narayanan R, Lee SH, Murata K, Hayashi Y (2007) The role of CaMKII as an F-actin-bundling protein crucial for maintenance of dendritic spine structure. Proc Natl Acad Sci U S A 104:6418–6423. 10.1073/pnas.0701656104 17404223 PMC1851051

[B52] Rivero-Gutiérrez B, Anzola A, Martínez-Augustin O, de Medina FS (2014) Stain-free detection as loading control alternative to Ponceau and housekeeping protein immunodetection in Western blotting. Anal Biochem 467:1–3. 10.1016/j.ab.2014.08.027 25193447

[B53] Rust MJ, Bates M, Zhuang X (2006) Sub-diffraction-limit imaging by stochastic optical reconstruction microscopy (STORM). Nat Methods 3:793–795. 10.1038/nmeth929 16896339 PMC2700296

[B54] Saito T, Matsuba Y, Yamazaki N, Hashimoto S, Saido TC (2016) Calpain activation in Alzheimer's model mice is an artifact of APP and presenilin overexpression. J Neurosci 36:9933–9936. 10.1523/JNEUROSCI.1907-16.2016 27656030 PMC5030353

[B55] Shankar GM, Li S, Mehta TH, Garcia-Munoz A, Shepardson NE, Smith I, Brett FM, Farrell MA, Rowan MJ, Lemere CA, Regan CM, Walsh DM, Sabatini BL, Selkoe DJ (2008) Amyloid-beta protein dimers isolated directly from Alzheimer's brains impair synaptic plasticity and memory. Nat Med 14:837–842. 10.1038/nm1782 18568035 PMC2772133

[B56] Spires TL, Meyer-Luehmann M, Stern EA, McLean PJ, Skoch J, Nguyen PT, Bacskai BJ, Hyman BT (2005) Dendritic spine abnormalities in amyloid precursor protein transgenic mice demonstrated by gene transfer and intravital multiphoton microscopy. J Neurosci 25:7278–7287. 10.1523/JNEUROSCI.1879-05.2005 16079410 PMC1820616

[B57] Spires-Jones TL, Meyer-Luehmann M, Osetek JD, Jones PB, Stern EA, Bacskai BJ, Hyman BT (2007) Impaired spine stability underlies plaque-related spine loss in an Alzheimer's disease mouse model. Am J Pathol 171:1304–1311. 10.2353/ajpath.2007.070055 17717139 PMC1988879

[B58] Star EN, Kwiatkowski DJ, Murthy VN (2002) Rapid turnover of actin in dendritic spines and its regulation by activity. Nat Neurosci 5:239–246. 10.1038/nn811 11850630

[B59] Steger C (1998) An unbiased detector of curvilinear structures. IEEE Trans Pattern Anal 20:113–125. 10.1109/34.659930

[B60] Tang AH, Chen H, Li TP, Metzbower SR, MacGillavry HD, Blanpied TA (2016) A trans-synaptic nanocolumn aligns neurotransmitter release to receptors. Nature 536:210–214. 10.1038/nature19058 27462810 PMC5002394

[B61] Terman JR, Kashina A (2013) Post-translational modification and regulation of actin. Curr Opin Cell Biol 25:30–38. 10.1016/j.ceb.2012.10.009 23195437 PMC3578039

[B62] Tsai J, Grutzendler J, Duff K, Gan WB (2004) Fibrillar amyloid deposition leads to local synaptic abnormalities and breakage of neuronal branches. Nat Neurosci 7:1181–1183. 10.1038/nn1335 15475950

[B63] van de Linde S, Löschberger A, Klein T, Heidbreder M, Wolter S, Heilemann M, Sauer M (2011) Direct stochastic optical reconstruction microscopy with standard fluorescent probes. Nat Protoc 6:991–1009. 10.1038/nprot.2011.336 21720313

[B64] Venkataramani V, Herrmannsdörfer F, Heilemann M, Kuner T (2016) SuReSim: simulating localization microscopy experiments from ground truth models. Nat Methods 13:319–321. 10.1038/nmeth.3775 26928761

[B65] Volianskis A, Køstner R, Mølgaard M, Hass S, Jensen MS (2010) Episodic memory deficits are not related to altered glutamatergic synaptic transmission and plasticity in the CA1 hippocampus of the APPswe/PS1deltaE9-deleted transgenic mice model of ss-amyloidosis. Neurobiol Aging 31:1173–1187. 10.1016/j.neurobiolaging.2008.08.005 18790549

[B66] Wilson RS, Beckett LA, Barnes LL, Schneider JA, Bach J, Evans DA, Bennett DA (2002) Individual differences in rates of change in cognitive abilities of older persons. Psychol Aging 17:179–193. 10.1037/0882-7974.17.2.179 12061405

[B67] Woo JA, Zhao X, Khan H, Penn C, Wang X, Joly-Amado A, Weeber E, Morgan D, Kang DE (2015) Slingshot-Cofilin activation mediates mitochondrial and synaptic dysfunction via Abeta ligation to beta1-integrin conformers. Cell Death Differ 22:921–934. 10.1038/cdd.2015.5 25698445 PMC4423195

[B68] Wu HY, Hudry E, Hashimoto T, Kuchibhotla K, Rozkalne A, Fan Z, Spires-Jones T, Xie H, Arbel-Ornath M, Grosskreutz CL, Bacskai BJ, Hyman BT (2010) Amyloid beta induces the morphological neurodegenerative triad of spine loss, dendritic simplification, and neuritic dystrophies through calcineurin activation. J Neurosci 30:2636–2649. 10.1523/JNEUROSCI.4456-09.2010 20164348 PMC2841957

[B69] Xu K, Zhong G, Zhuang X (2013) Actin, spectrin, and associated proteins form a periodic cytoskeletal structure in axons. Science 339:452–456. 10.1126/science.1232251 23239625 PMC3815867

[B70] Zhao L, Ma QL, Calon F, Harris-White ME, Yang F, Lim GP, Morihara T, Ubeda OJ, Ambegaokar S, Hansen JE, Weisbart RH, Teter B, Frautschy SA, Cole GM (2006) Role of p21-activated kinase pathway defects in the cognitive deficits of Alzheimer disease. Nat Neurosci 9:234–242. 10.1038/nn1630 16415866

