# Supplementary figures and images for: Aβ mediates F-actin disassembly in dendritic spines leading to cognitive deficits in Alzheimer's disease
Source: J Neurosci. 2018 Jan 31;38(5):1085–99. doi: 10.1523/JNEUROSCI.2127-17.2017 (PMC5792472; doi:10.1523/JNEUROSCI.2127-17.2017)

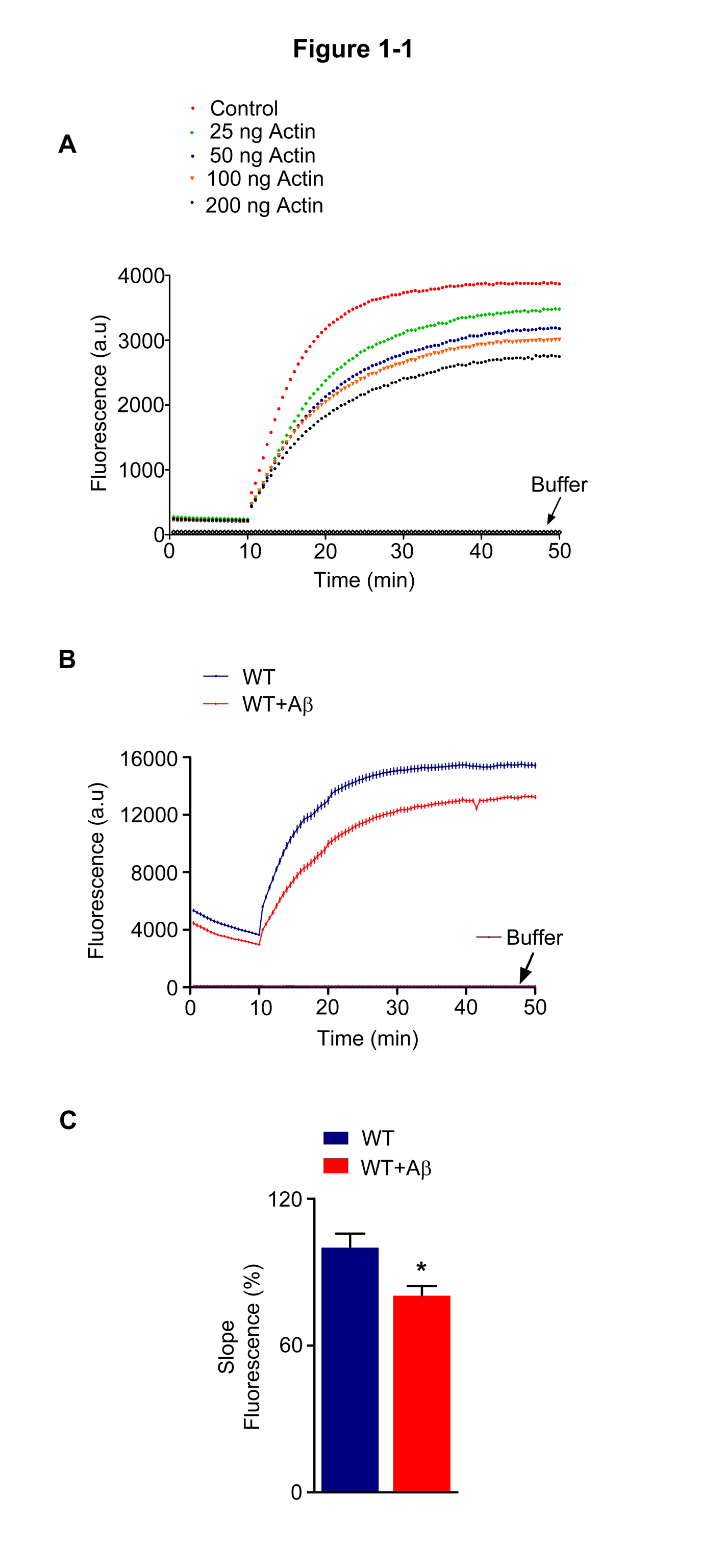

Supplement: Figure 1-1 [file zns999170462so1.tif]

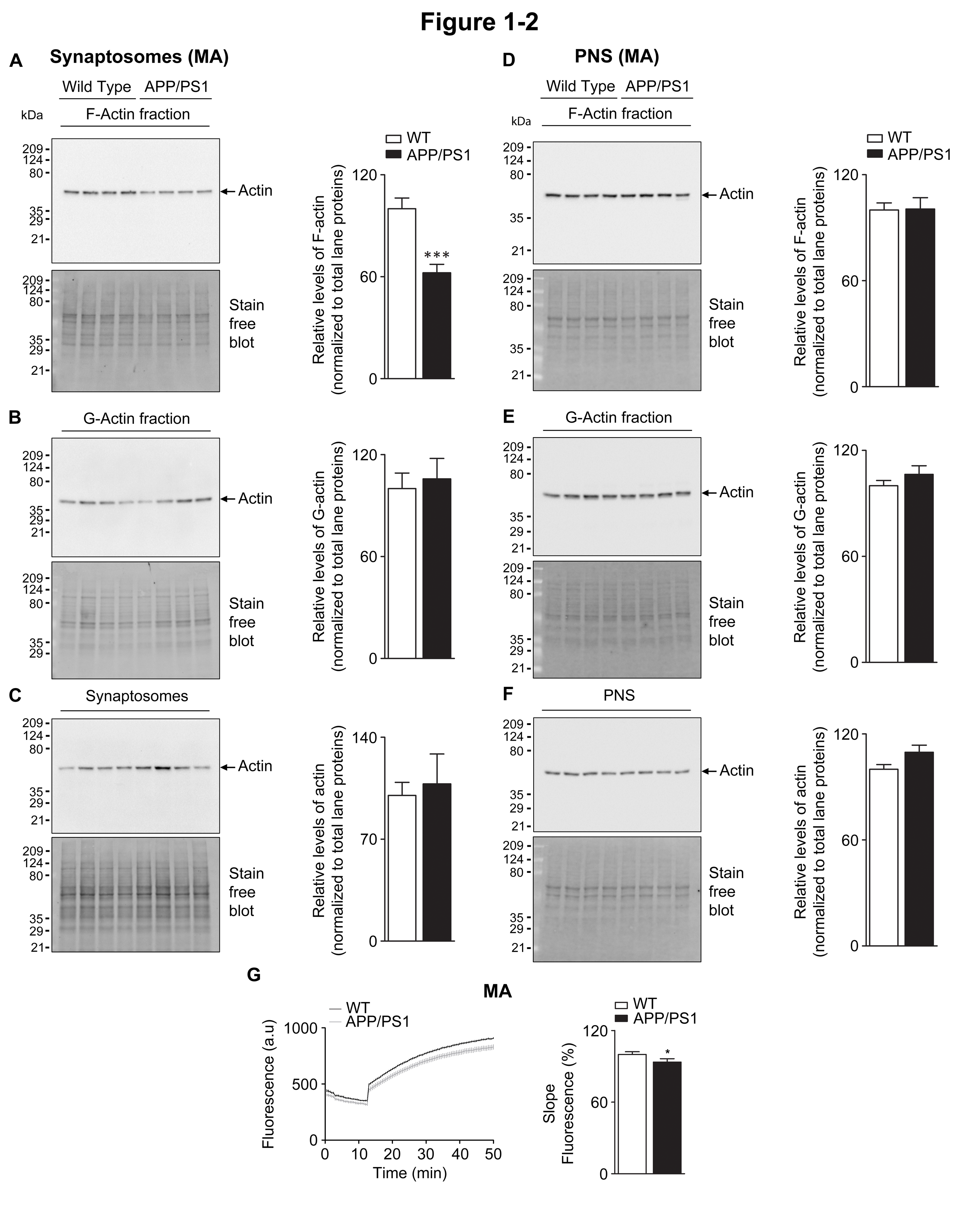

Supplement: Figure 1-2 [file zns999170462so2.tif]

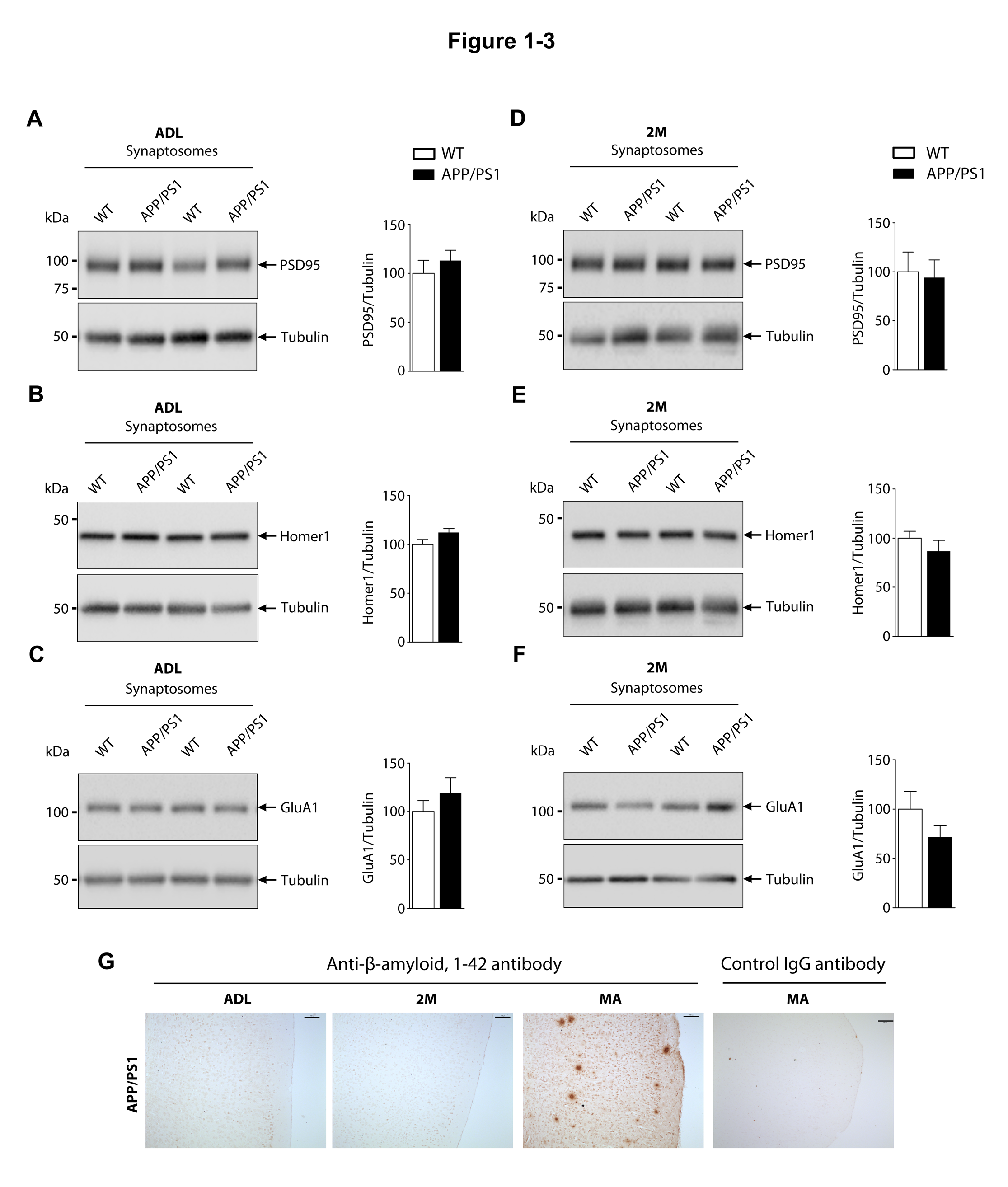

Supplement: Figure 1-3 [file zns999170462so3.tif]

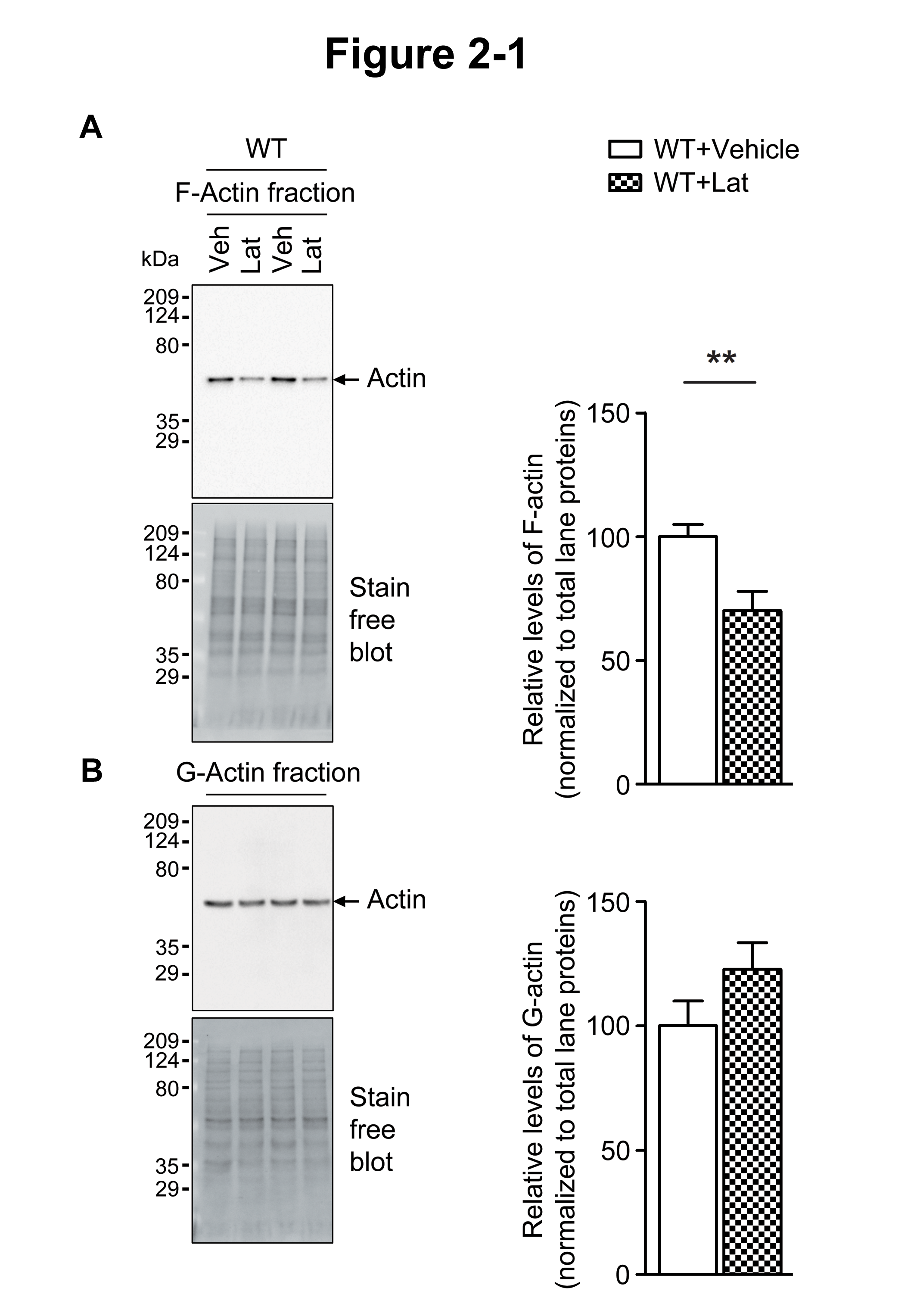

Supplement: Figure 2-1 [file zns999170462so4.tif]

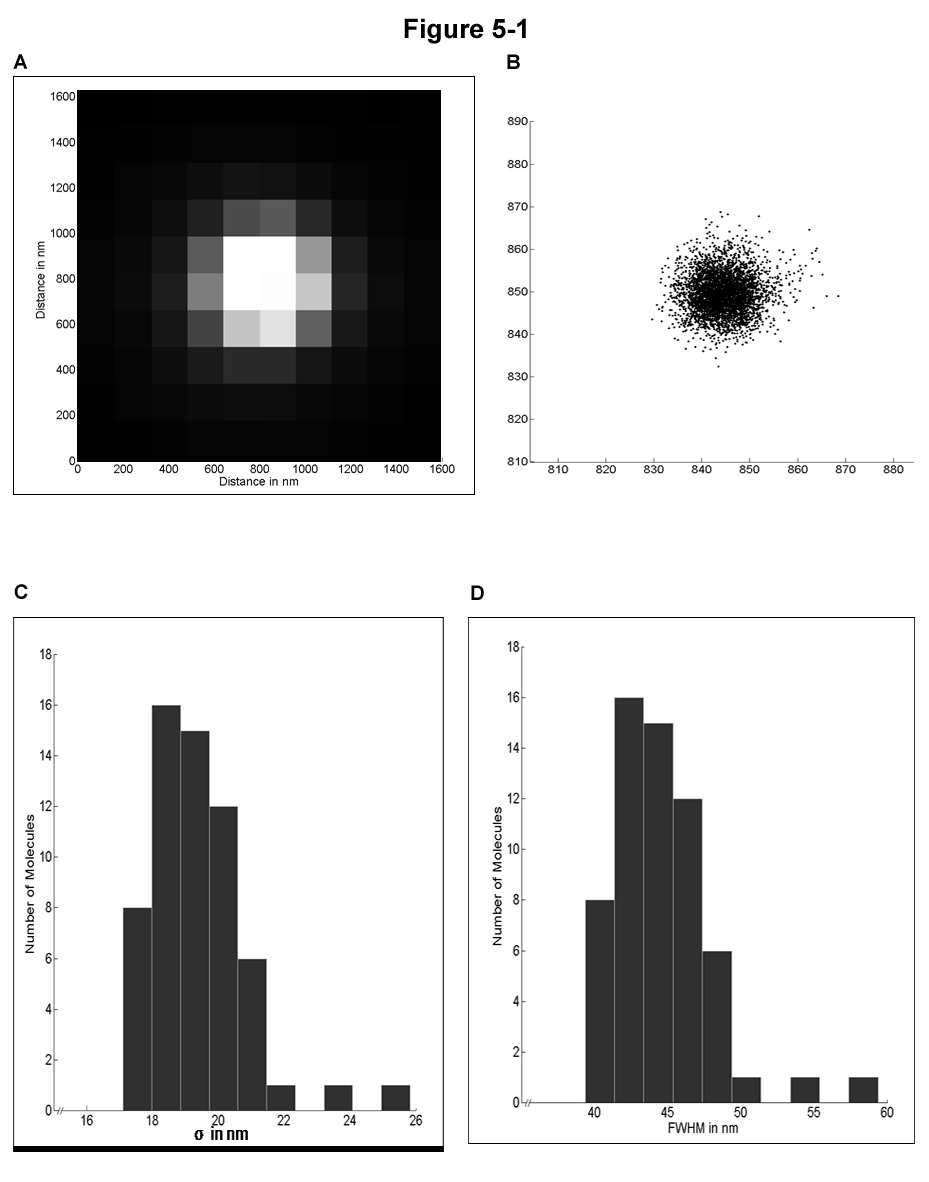

Supplement: Figure 5-1 [file zns999170462so5.tif]

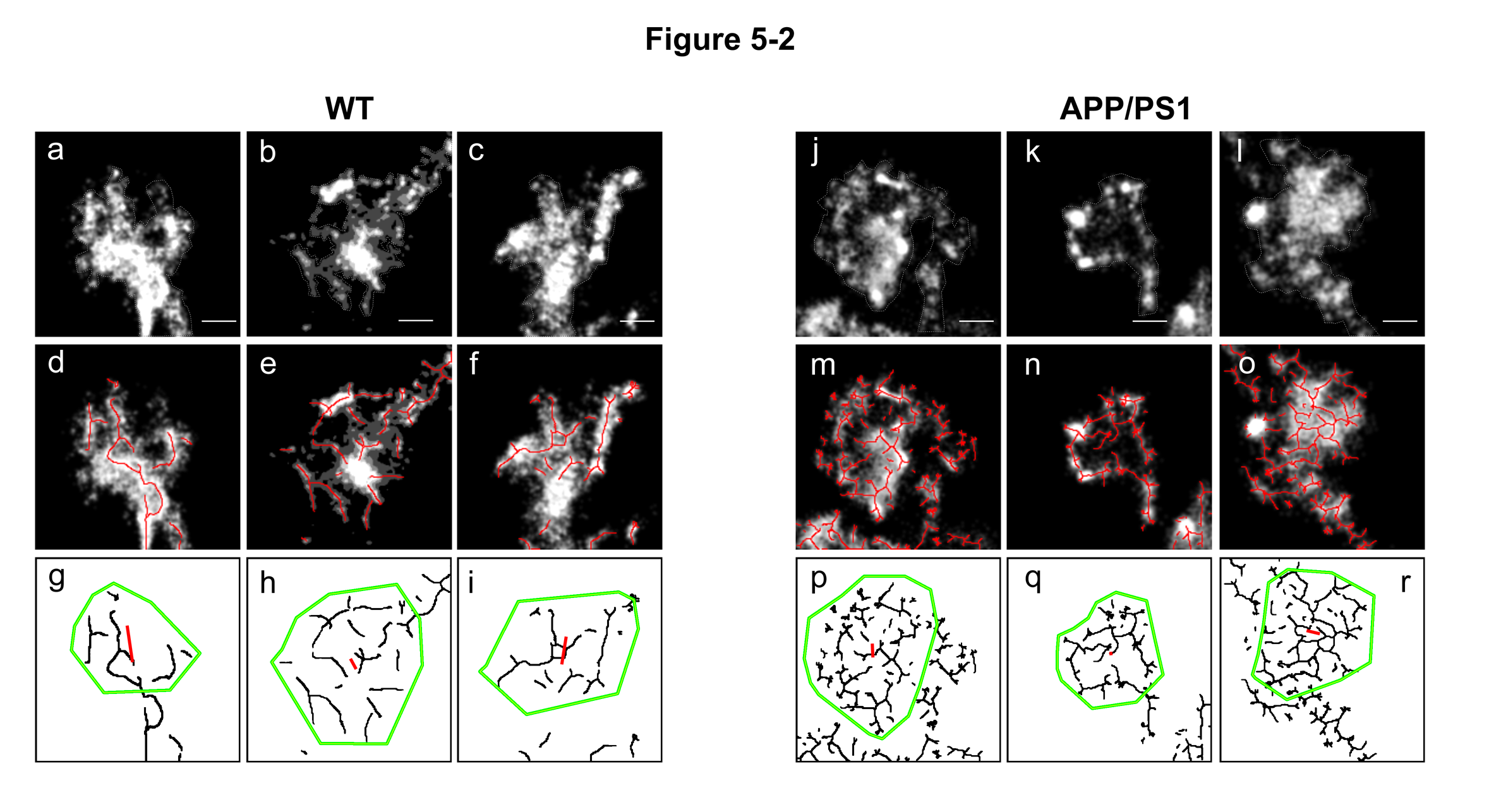

Supplement: Figure 5-2 [file zns999170462so6.tif]

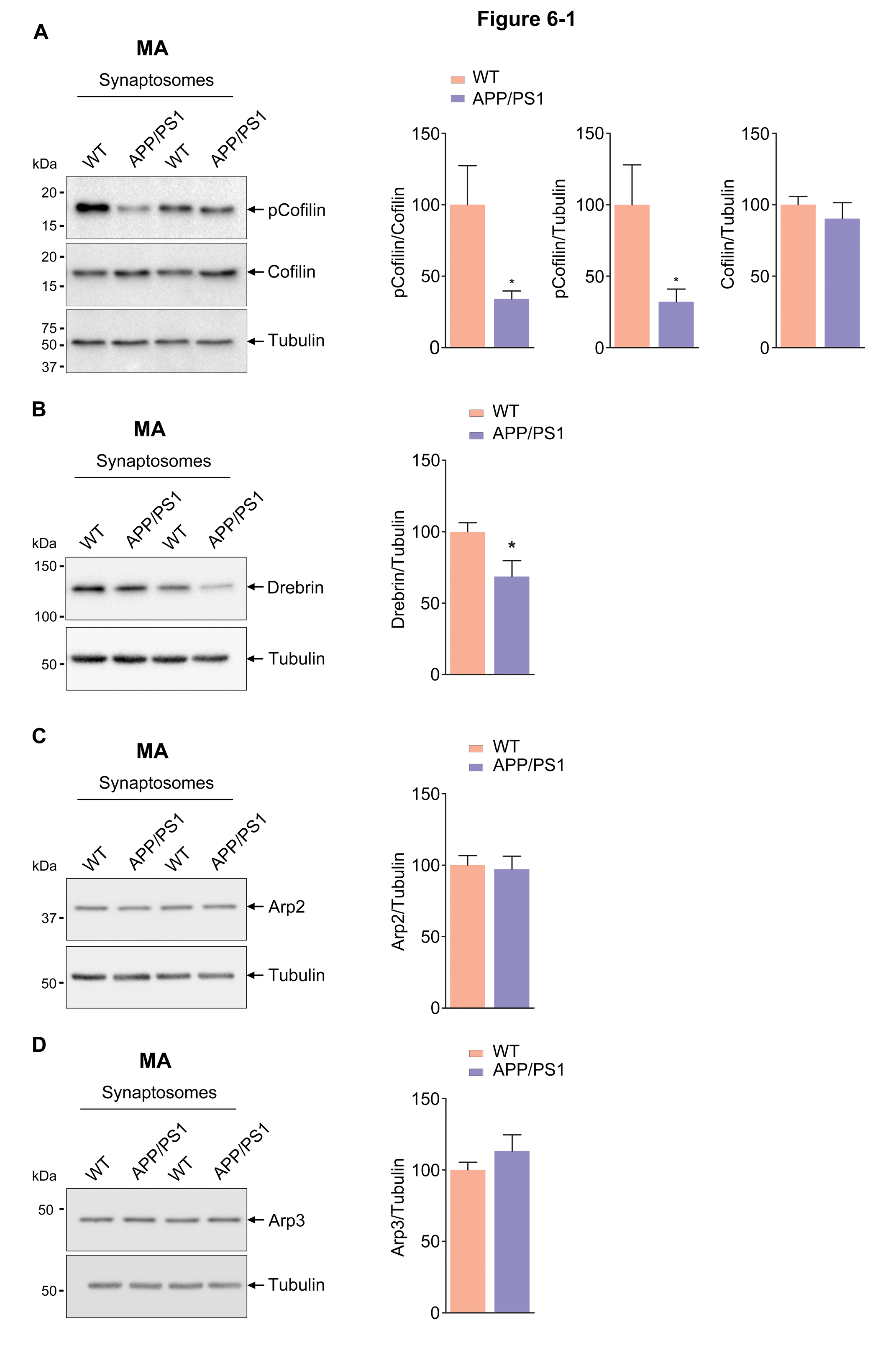

Supplement: Figure 6-1 [file zns999170462so7.tif]
